# Open-Label Placebos as Adjunct for the Preventive Treatment of Migraine: A Randomized Clinical Trial

**DOI:** 10.1001/jamanetworkopen.2025.35739

**Published:** 2025-10-08

**Authors:** Julian Kleine-Borgmann, Katharina Schmidt, Linda Ludwig, Moritz Schulz, Dagny Holle-Lee, Charly Gaul, Ulrike Bingel

**Affiliations:** 1 Center for Translational Neuro- and Behavioral Sciences, Department of Neurology, University Hospital Essen, Essen, Germany; 2 Headache Center Frankfurt, Frankfurt am Main, Germany

## Abstract

**Importance:**

Placebo effects contribute substantially to the therapeutic success of many treatments, particularly in pain-related conditions. Open-label placebos (OLPs) offer an ethically acceptable approach to harness this potential without deception.

**Objective:**

To evaluate the efficacy of a 3-month OLP regimen in reducing headache and migraine days and improving migraine-related outcomes, including medication use, disability, and quality of life, in patients with migraine.

**Design, Setting, and Patients:**

This controlled, bicenter, parallel-group randomized clinical trial, with a 3-month treatment phase, enrolled adults with episodic or chronic migraine between November 9, 2020, and November 1, 2022. The trial was conducted at 2 tertiary headache centers in Germany (University Medicine Essen and Headache Center Frankfurt).

**Interventions:**

Participants received OLPs plus treatment as usual (TAU) or TAU alone. OLPs were administered twice daily for 3 months.

**Main Outcomes and Measures:**

The preregistered primary outcome was the change in monthly headache days from a baseline to a test period after 3 months. Secondary outcomes included patient-reported quality of life assessed using the 12-Item Short-Form Health Survey physical component summary, pain-related disability assessed using the Pain Disability Index and Headache Impact Test, and global improvement.

**Results:**

Of the 120 patients (median age, 34.2 years; 95% CI, 29.8-39.3 years; 103 [86%] female), 102 (85%) had episodic migraine, and 18 (15%) had chronic migraine. All participants completed the study. Headache days did not significantly decrease in the OLP group compared with TAU. Similarly, there was no difference in the number of migraine days, pain intensity, days of rescue medication, and the 50% responder rate. However, OLP-treated patients reported improved quality of life (β = 4.25; 95% CI, 1.33-7.17; *d* = 0.47; *P* = .01), reduced pain-related disability (Pain Disability Index: β = −5.96; 95% CI, −9.01 to −2.92; *d* = 0.53; *P* < .001; Headache Impact Test 6: β = −1.88; 95% CI, −3.28 to −0.48; *d* = 0.35; *P* = .02), and higher global improvement (χ^2^ = 14.16; *P* = .01) compared with TAU patients.

**Conclusions and Relevance:**

In this randomized clinical trial, OLP treatment did not reduce headache frequency but was associated with improvements in quality of life and pain-related disability. Future research should clarify the mechanisms underlying these effects and determine their potential supportive role in migraine care for selected patients.

**Trial Registration:**

drks.de identifier: DRKS00021259

## Key Points

**Question:**

Can open-label placebos (OLPs), administered without deception, reduce headache days or improve related outcomes in patients with migraine?

**Findings:**

In this randomized clinical trial, a 3-month OLP regimen did not reduce monthly headache days (the primary outcome) or migraine days (a secondary outcome). Patients receiving OLPs did experience significant improvements in the secondary outcomes of pain-related disability, quality of life, and patient-reported global improvement.

**Meaning:**

The results of this study suggest that OLPs might have a supportive role in migraine care for selected patients, compared with those receiving treatment as usual.

## Teaser

This randomized clinical trial of patients with migraine assesses whether an open-label placebo plus treatment as usual compared with treatment as usual alone is effective in treating monthly headache days.

## Introduction

Headache disorders rank as the third leading cause of disability across all age groups, with migraine contributing substantially to both individual impairment and socioeconomic burden.^1^ As the most common neurologic condition, migraine affects approximately 15% of the global population.^2^ Migraine diagnosis follows the *International Classification of Headache Disorders, Third Edition (ICHD-3)* criteria.^3^ Immediate treatment options include nonsteroidal anti-inflammatory drugs, triptans, calcitonin gene–related peptide (CGRP) antagonists (gepants), and lasmiditan. Preventive therapy, which is recommended for frequent, disabling, or treatment-resistant migraine attacks, menstrual migraine, or risk of medication overuse, includes lifestyle interventions (regular sleep, meals, exercise, relaxation, and stress management) and medications such as antidepressants, β-blockers, antiseizure drugs, CGRP antagonists, monoclonal antibodies targeting CGRP, and onabotulinumtoxin A.^4,5,6,7^ Adverse effects often limit the use of antidepressants and antiseizure medications.^8,9^ Additionally, prescription guidelines and resource availability limit access to novel pharmacologic treatments (eg, CGRP antagonists and onabotulinumtoxin A) and nonpharmacologic strategies (eg, inpatient multimodal programs).

Clinical and mechanistic studies highlight substantial placebo effects in acute migraine treatments, with placebo response rates as high as 46% in trials.^10,11^ Moreover, a meta-analysis of 21 anti-CGRP antibody studies indicated that placebo effects may explain up to 66% of therapeutic improvements.^12^ Specifically, the efficacy and safety trial of the intravenously administered CGRP-antibody eptinezumab reported that up to 74% of observed benefits may be due to placebo responses.^13^ Despite the clinical importance of placebo effects, ethical concerns, such as avoiding deception, limit their therapeutic use.^14^

Randomized clinical trials (RCTs) have shown that open-label placebos (OLPs) (ie, placebos given openly) can produce clinically meaningful improvements in various conditions, especially acute and chronic pain.^15,16,17,18,19,20,21^ In a drug-labeling study, Kam-Hansen et al^22^ highlighted the efficacy of OLPs vs no treatment for acute migraine attacks.

This RCT, conducted at 2 German tertiary headache centers, assessed the effects of a 3-month OLP treatment plus treatment as usual (TAU) compared with TAU alone on monthly headache days (MHDs; primary end point). Following current guidelines for migraine prevention trials, to our knowledge, this study represents the longest OLP RCT conducted to date.^23^ Secondary end points included changes in migraine days, mean pain intensity, rescue medication use, global impression of change, quality of life, pain-related disability, and OLP tolerability.

## Methods

### Trial Population

Adults 18 years or older with a history of episodic or chronic migraine based on self-report and diagnosed by a specialist in neurology following the *ICHD-3* criteria^3^ for 12 months or more who had at least 4 migraine days per month in the 3 months before screening were eligible for trial participation. Race and ethnicity were not assessed because this trial was conducted in Germany, where such demographic information is not routinely collected in clinical trials. Eligibility was confirmed during a 4-week baseline period in which patients monitored and reported headache days using a standardized symptom diary. The concurrent use of any migraine prevention medication maintained at a stable dose (starting 3 months before randomization) and/or nonpharmacologic preventive strategies was permitted. Exclusion criteria were substance or alcohol abuse, comorbid major depression, schizophrenia, suicidality, hypersensitivity or allergy to any ingredient of the OLP pills, participation in other studies using investigational drugs within the 3 months before inclusion, any pain condition apart from migraine, as well as pregnancy or breastfeeding in women. Patients were recruited through clinical practices, advertisements, and referrals.

The ethics committees at each trial center approved the trial protocol. The trial was conducted following the Declaration of Helsinki.^24^ It was preregistered at the German Clinical Trials Register on October 9, 2020, and the protocol was published before trial initiation.^25^ Written informed consent was obtained, and patients were financially compensated (€145). This study followed the Consolidated Standards of Reporting Trials (CONSORT) 2010 reporting guideline.^26^ The trial protocol can be found in Supplement 1.

### Trial Design

This single-blinded, bicenter RCT with a parallel-group, between-participant design, including patients with episodic and chronic migraine, was conducted at 2 tertiary headache centers, the Department of Neurology, University Hospital Essen, and the Headache Center Frankfurt, between November 9, 2020, and November 1, 2022. The trial encompassed up to 6 visits: visit 0: eligibility, informed consent, and 1-month baseline period; visit 1: baseline assessments and randomization; visit 2: interim assessment; visit 3: 1-month test period; visit 4: follow-up; visit X: optional magnetic resonance imaging; see eMethods and eFigure 1 in Supplement 2 for details of the study design. Magnetic resonance imaging and analyses of personality and biological factors will be reported separately.

At the end of the 4-week baseline period, all patients were instructed about OLPs based on a video sequence (see the trial protocol in Supplement 1). Patients were randomized (1:1) to receive either OLPs plus TAU or TAU alone for 3 months, with randomization stratified by center using a schedule generated in R, version 4.5.0 (R Foundation for Statistical Computing) by a person not otherwise involved in the study. Due to the nature of OLP, patients were not blinded, but site personnel and data analysts remained unaware of group assignments. Patients were instructed to keep their allocation confidential to maintain blinding.

### Treatment

Patients in both groups received a cardboard box containing a labeled dispenser with 168 placebo tablets (P Tabletten, Zentiva Pharma GmbH), instructions explicitly stating that the tablets had no active ingredients, and directions to take 1 pill twice daily for 3 months alongside TAU (OLP group), or a note indicating assignment to the control group requiring no further action (TAU group). The white OLP tablets included lactose monohydrate, cellulose, magnesium stearate, and microcrystalline cellulose. The OLP regimen was based on previous studies.^16,17^ Patients documented daily tablet intake in a headache diary.

### Trial Assessment and End Points

Assessments were performed via online questionnaires (LimeSurvey, LimeSurvey GmbH) and paper-based pain diaries following International Headache Society guidelines for migraine prevention trials.^23^ The primary end point was the change in moderate-to-severe MHDs from a 4-week baseline (visit 1) to a 4-week test period (visit 3). Secondary end points included changes in monthly migraine days (MMDs), mean pain intensity (11-point numeric rating scale, with 0 indicating no pain and 10 indicating unbearable pain), rescue medication days, disability (Pain Disability Index [PDI]^27^ and Headache Impact Test 6 [HIT-6] scores^28^), global impression of change (Patient Global Impression of Change [PGIC]^29^), quality of life (12-Item Short-Form Health Survey [SF-12] score^30^), and tolerability (General Assessment of Side Effects [GASE] score^31,32^). The 50% responder rate and changes at interim (visit 2) and follow-up (visit 4) were also analyzed. An MHD was defined as a day with moderate or severe pain that lasted at least 4 hours or a day with a headache lasting at least 30 minutes that was successfully treated by headache medication. An MMD was defined as a day with a headache that lasted at least 4 hours and met *ICHD-3* criteria C and D for migraine without aura, criteria B and C for migraine with aura, or *ICHD-3* criteria for probable migraine or a day with a headache that was successfully treated with a triptan, ergotamine, or other migraine-specific medication. Further exploratory end points (not reported here) are detailed in the study protocol.^25^

### Statistical Analysis

All randomized patients were included in the analysis. The sample size was calculated to achieve 90% power (α = .05, effect size *f* = 0.2, *d* = 0.4) for the primary end point.^17^ To account for 10% dropout, 150 patients (75 per group) were targeted. For count outcomes (ie, headache, migraine, and rescue medication days), generalized linear mixed-effects models assuming a Poisson distribution were fitted using maximum likelihood estimation (Nelder-Mead optimizer), without imputation of missings, and included group, time, and their interaction as fixed effects and patient identification as a random effect. For continuous outcomes (ie, mean pain intensity, SF-12 score, HIT-6 score, and PDI score), robust linear mixed-effects models were used. Residual diagnostics are provided in eTables 1 to 18 in Supplement 2. Group differences in PGIC response and 50% responder rates were tested per visit using Pearson χ^2^ tests with Yates correction. A Wilcoxon rank-sum test was used to detect group differences in medication-attributed symptoms. A 2-sided α = .05 was applied. All analyses were performed using R, version 4.5.0 and RStudio, version 2025.5.0.496 (RStudio). False discovery rate correction was applied for changes from baseline to the test period, and 95% CIs were estimated using bootstrapping. Analyses for visits 2 and 4 were exploratory. A data monitoring committee was not established due to the favorable safety profile of OLPs and the low associated risk.^16,17,33,34^

## Results

**Figure 1.**
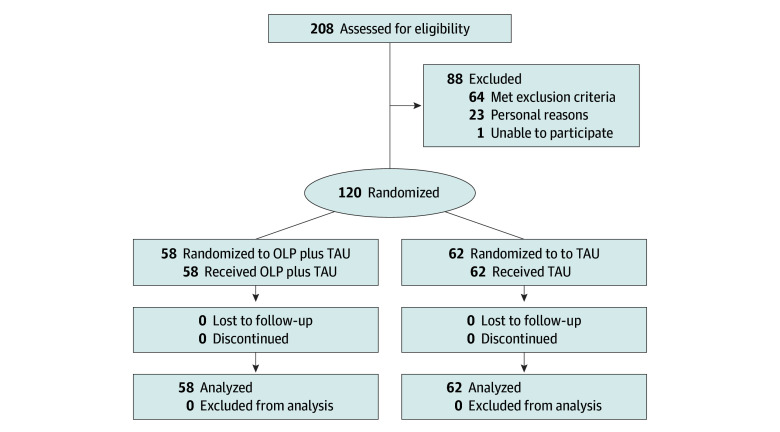
Patient Flow Chart Data are from University Hospital Essen and Headache Center Frankfurt. OLP indicates open-label placebo; TAU, treatment as usual.

A total of 120 patients (median age, 34.2 years; 95% CI, 29.8-39.3 years; 103 [86%] female and 17 [14%] male), 58 in the OLP group and 62 in the TAU group, participated in the trial. Patients had either episodic (102 [85%]) or chronic (18 [15%]) migraine. No patients were lost to follow-up or excluded (Figure 1). Due to COVID-19–related constraints on the trial, including office and laboratory closures, health care prioritization, and recruitment halts, we were unable to reach the targeted sample size of 150 patients. Most enrollments (102 [85%]) were conducted at University Hospital Essen. Nevertheless, both centers maintained a balanced OLP-TAU randomization ratio, with Headache Center Frankfurt at 50:50 and University Hospital Essen at 48:52. Table 1 lists the sample characteristics.

**Table 1.** Baseline Characteristics of the Study Patients^a^

Characteristic	No. (%) of patients^b^	
	OLP (n = 58)	TAU (n = 62)
Demographic characteristics		
Sex		
Female	49 (84)	54 (87)
Male	9 (16)	8 (13)
Age, median (95% CI), y	34.2 (29.8-39.3)	32.6 (26.8-42.6)
BMI, median (95% CI)	24.3 (23.0-26.0)	24.0 (22.6-25.4)
Length of education, median (95% CI), y	15.0 (13.0-15.0)	16.0 (15.0-16.0)
History and migraine characteristics		
Any concomitant condition	43 (74)	44 (71)
Migraine type		
Chronic migraine	8 (14)	10 (16)
Episodic migraine	50 (86)	52 (84)
Time since migraine onset, y		
>5	50 (86)	56 (90)
0.5-1	0	0
1-2	3 (5)	0
2-5	5 (9)	6 (10)
Prior GP consultation	43 (74)	48 (77)
Prior neurologic consultation	44 (76)	50 (81)
Concomitant pharmacologic preventive treatment	18 (31)	23 (37)
Concomitant nonpharmacologic preventive treatment	54 (93)	57 (92)
Lost work days, median (95% CI) (3 months before visit 1)	3.5 (1.0-6.0)	4.0 (1.5-5.0)

Abbreviations: BMI, body mass index (calculated as weight in kilograms divided by height in meters squared); GP, general practitioner; OLP, open-label placebo; TAU, treatment as usual.

^a^ All characteristics are based on the baseline data collected at visit 0, visit 1, or during the baseline period. For outcome baseline values, see Table 2.

^b^ Unless otherwise indicated.

### Primary End Point: MHDs

**Figure 2.**
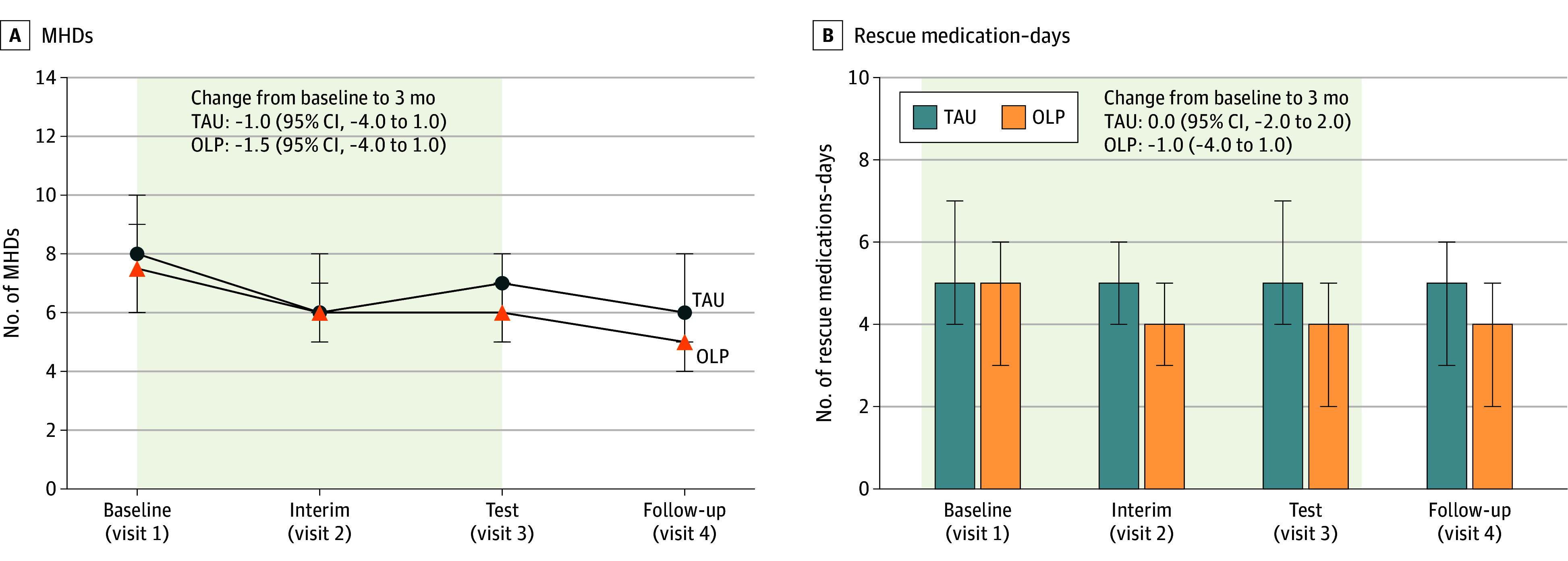
Monthly Headache- and Rescue Medication–Days Median monthly headache days (MHDs) (primary end point) and rescue medication–days in the past 4-week period and bootstrapped 95% CIs (error bars) are shown per group. Baseline was 1 month before randomization; interim, randomization plus 1 month; test, randomization plus 3 months; and follow-up, randomization plus 6 months. OLP indicates open-label placebo group; TAU, treatment-as-usual group.

During the test period, patients in the OLP group had a median of 6.0 (95% CI, 5.0-7.0) MHDs compared with 7.0 (95% CI, 5.0-8.0) MHDs in the TAU group, which was not a significant reduction (Table 2 and Figure 2A; eTable 1 in Supplement 2). Exploratory analysis from baseline to follow-up revealed a significant main effect of time, indicating fewer MHDs for both groups at the 6-month follow-up compared with baseline (incidence rate ratio [IRR], 0.84; 95% CI, 0.74-0.95; *P* = .005). Again, no significant between-group differences were found (eTable 2 in Supplement 2).

**Table 2.** Primary and Secondary End Point Results

End point	OLP (n = 58)	TAU (n = 62)	Group difference (95% CI)^a^	Statistical result	Adjusted *P*^b^
MHDs, median (95% CI) (primary end point)					
Baseline	7.5 (6.0 to 9.0)	8.0 (6.0 to 10.0)	NA	NA	NA
Test	6.0 (5.0 to 7.0)	7.0 (5.0 to 8.0)	−1.0 (−2.5 to 1.0)	IRR, 0.89; 95% CI, 0.75 to 1.07	.34
MMDs, median (95% CI) (secondary end point)					
Baseline	6.0 (5.0 to 8.0)	8.0 (6.0 to 9.0)	NA	NA	NA
Test	5.0 (4.0 to 7.0)	6.0 (5.0 to 7.0)	−1.0 (−3.0 to 1.0)	IRR, 0.93; 95% CI, 0.77 to 1.12	.46
Pain intensity, mean (95% CI) (secondary end point)					
Baseline	4.7 (4.7 to 5.6)	4.8 (4.5 to 5.3)	NA	NA	NA
Test	5.2 (4.8 to 5.8)	5.7 (4.9 to 5.8)	−0.4 (−0.8 to 0.4)	β = −0.17; 95% CI, −0.58 to 0.24	.70
Rescue medication–days, median (95% CI)					
Baseline	5.0 (3.0 to 6.0)	5.0 (4.0 to 7.0)	NA	NA	NA
Test	4.0 (2.0 to 5.0)	5.0 (4.0 to 7.0)	−1.0 (−4.0 to 0.0)	IRR, 0.83; 95% CI, 0.67 to 1.04	.25
Patient Global Impression of Change, No. (%)^c^					
Test	27 (46.6)	15 (24.2)	NA	χ^2^ = 14.16	.01
SF-12 mental component summary score, median (95% CI)					
Baseline	49.4 (44.6 to 51.8)	47.4 (43.3 to 52.4)	NA	NA	NA
Test	49.5 (45.3 to 53.1)	46.7 (42.8 to 50.5)	2.8 (−2.5 to 8.3)	β = 2.72; 95% CI, −0.24 to 5.68	.12
SF-12 physical component summary score, median (95% CI)					
Baseline	36.5 (34.1 to 39.6)	38.0 (34.1 to 43.4)	NA	NA	NA
Test	41.3 (39.0 to 47.2)	39.1 (36.7 to 42.8)	2.21 (−1.78 to 8.38)	β = 4.25; 95% CI, 1.33 to 7.17	.01
Pain Disability Index, median (95% CI)					
Baseline	22.0 (19.0 to 24.0)	20.5 (17.0 to 24.0)	NA	NA	NA
Test	15.0 (12.0 to 19.0)	20.0 (16.0 to 28.0)	5.0 (13.5 to 1.0)	β = 5.96; 95% CI, −9.01 to −2.92	<.001
Headache Impact Test 6, median (95% CI)					
Baseline	64.0 (63.0 to 65.0)	64.5 (64.0 to 66.0)	NA	NA	NA
Test	62.0 (61.0 to 64.0)	65.0 (64.0 to 66.0)	−3.0 (−4.0 to 0.0)	β = −1.88; 95% CI, −3.28 to −0.48	.02
Medication-attributed symptom count, mean (95% CI)	0.5 (0.2 to 1.4)	0.1 (−0.1 to 0.1)	0.4 (−0.5 to 1.3)	W = 2014	.01
≥50% Responder rate, No. (%)	16 (27.6)	16 (25.8)	0 (1.8)	χ^2^ = 0.01	.94

Abbreviations: IRR, incidence rate ratio; MHDs, monthly headache days; MMDs, monthly migraine days; NA, not applicable; OLP, open-label placebo; SF-12, 12-Item Short Form Health Survey; TAU, treatment as usual.

^a^ IRRs were calculated based on generalized linear mixed-effects models assuming a Poisson distribution. β Estimates represent results of robust linear mixed-effects models.

^b^ *P* values have been adjusted for multiple comparisons by false discovery rate correction.

^c^ Proportion of items indicating improvement (ie, improved, much improved, and very much improved).

### Secondary End Points

#### Monthly Migraine Days 

No significant between-group differences in change from the baseline to the test period were observed. However, the exploratory analysis of all time points revealed a statistically significant reduction in MMDs over time in both groups (visit 2: IRR, 0.87; 95% CI, 0.77-0.99; *P* = .03; visit 3: IRR, 0.88; 95% CI, 0.77-1.00; *P* = .04; visit 4: IRR, 0.82; 95% CI, 0.71-0.93; *P* = .002) (eFigure 2, eTable 3, and eTable 4 in Supplement 2).

#### Mean Pain Intensity

After 3 months, both groups showed a significant increase in mean pain intensity (main effect of time; β = 0.43; 95% CI, 0.14-0.71; *d* = 0.55; *P* = .009) (eTable 5 in Supplement 2). This effect persisted until visit 4 in our exploratory analysis. However, changes in pain intensity did not significantly differ between groups (eFigure 3, eTable 5, and eTable 6 in Supplement 2).

#### Rescue Medication Days

During the test period, patients receiving OLP in addition to TAU reported a median of 4 rescue medication days (95% CI, 2.0-5.0 days) compared with 5 days (95% CI, 4.0-7.0 days) in the TAU group, indicating no significant difference in change from baseline. Additionally, exploratory analyses revealed no group differences at any other visit (Figure 2B and eTables 7-10 in Supplement 2).

#### Global Impression of Change 

Patients in the OLP group reported significantly higher positive response rates (very much improved, much improved, and improved) compared with TAU after 1 month (χ^2^_5_ = 12.42; *P* = .03) and 3 months (χ^2^_5_ = 14.16; *P* = .02). At 6 months, differences were no longer significant (eFigure 4 in Supplement 2).

#### Quality of Life

**Figure 3.**
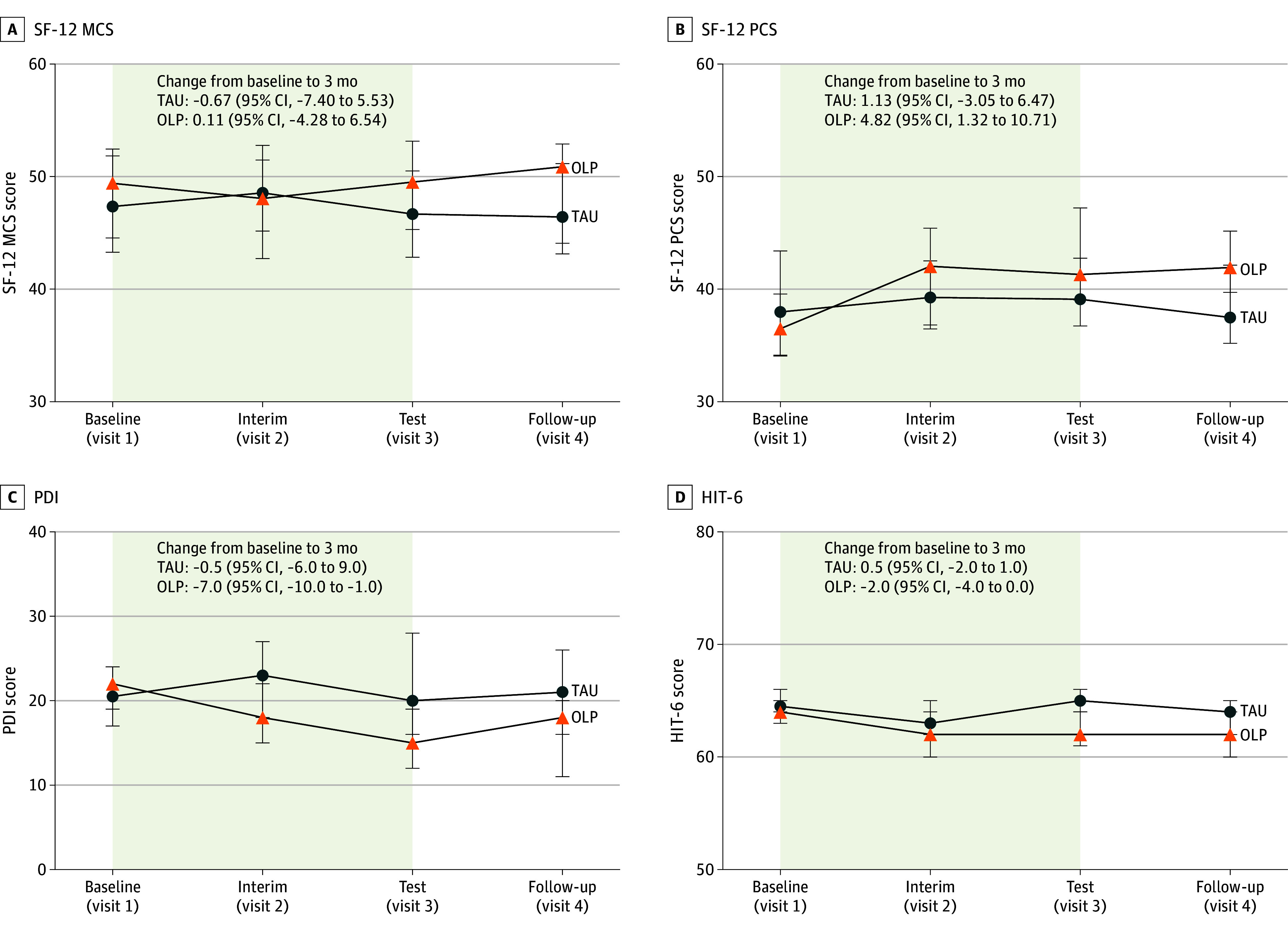
Quality of Life and Disability Changes in secondary outcome measures from the baseline (visit 1) to the test period (visit 3) in the treatment-as-usual (TAU) and open-label placebo (OLP) groups. Panels show estimated median scores with bootstrapped 95% CIs for the mental component summary (MCS) and physical component summary (PCS) scores of the 12-Item Short-Form Health Survey (SF-12), the Pain Disability Index (PDI), and the Headache Impact Test 6 (HIT-6). Baseline was 1 month before randomization; interim, randomization plus 1 month; test, randomization plus 3 months; and follow-up, randomization plus 6 months.

Neither the primary nor the exploratory analysis revealed a statistically significant difference in SF-12 mental component summary (MCS) scores between groups after 3 or 6 months (Figure 3A, eTable 11, and eTable 12 in Supplement 2). Regarding physical health, OLP-treated patients showed a significant increase in SF-12 physical component summary (PCS) scores during 3 months compared with patients who received TAU only (β = 4.25; 95% CI, 1.33-7.17; *d* = 0.47; *P* = .01) (Figure 3B and eTable 13 in Supplement 2). This difference remained significant at the 6-month follow-up (β = 3.37; 95% CI, 0.59-6.15; *d* = 0.39; *P* = .02) (eTable 14 in Supplement 2).

#### Pain-Related Disability

Patients who received OLP and TAU showed significantly greater improvements in PDI scores after 3 months compared with patients who received TAU alone (β = −5.96; 95% CI, −9.01 to −2.92; *d* = 0.53; *P* < .001) Figure 3C and eTable 15 in Supplement 2). Exploratory analysis revealed that this difference was observable after 1 month and persisted up to 6 months (visit 2: β = −3.86; 95% CI, −6.84 to 0.88; *d* = 0.32; *P* = .01; visit 4: β = −4.40; 95% CI, −7.38 to −1.41; *d* = 0.36; *P* = .004) (eTable 16 in Supplement 2). Moreover, OLP-treated patients experienced a significant reduction in HIT-6 scores after 3 months compared with TAU patients (β = −1.88; 95% CI, −3.28 to −0.48; *d* = 0.35; *P* = .02) (Figure 3D and eTable 17 in Supplement 2). This difference was not significant at 1 or 6 months (eTable 18 in Supplement 2).

#### Symptoms Attributed to Medication

After 3 months, the OLP group reported significantly more medication-related adverse effects compared with the TAU group (W = 2014; *P* = .01), although the overall symptom count was low. Specifically, 8 patients (14%) in the OLP group and 1 patient (2%) in the TAU group reported at least 1 symptom, including xerostomia, vertigo, gastrointestinal symptoms, hyperhidrosis, hot flushes, and insomnia.

#### 50% Responder Rate

After 3 months, 16 patients (29%) in the OLP group and 16 (26%) in the TAU group achieved a 50% or greater reduction in headache days, with no significant difference between groups (χ^2^ = 0.01; *P* = .94). At 6 months, the numbers remained similar (14 [29%] in the OLP group and 15 [25%] in the TAU group; χ^2^ = 0.05; *P* = .83).

## Discussion

This RCT evaluated the efficacy of a 3-month OLP treatment for migraine prevention in 120 patients, with 58 allocated to the OLP group and no loss to follow-up. Notably, based on patients’ individual decisions, 40 received OLP as a standalone intervention without concurrent preventive pharmacologic treatment.

The OLP treatment did not reduce the number of headache or migraine days or influence the 50% responder rate or mean pain intensity. Across both groups, headache and migraine days decreased slightly, whereas mean pain intensity increased, possibly due to increased self-awareness. The relatively high 50% responder rates in both groups may be explained by the high standard of usual care at tertiary centers. In contrast to these negative findings, OLP-treated patients showed improvements in quality of life, physical and mental health, and disability compared with patients receiving TAU only. Moreover, they reported significantly more global improvement (PGIC), a well-recognized patient-reported outcome recommended for use in chronic pain trials, with effects emerging after only 1 month.^35,36^

Migraine is associated with reduced quality of life, reflected in lower SF-12 mental (MCS) and physical (PCS) health scores compared with healthy populations.^37,38^ OLP-treated patients showed a 4.5-point gain on the PCS, which persisted through follow-up. Minimally clinically important differences for patients with migraine have not yet been established. However, an improvement of greater than 3.29 points on the PCS indicates clinically relevant changes in other pain conditions.^39^ Disability also improved, with a 2-point median reduction in headache-related disability (HIT-6) after 3 months, remaining stable at follow-up. Notably, baseline PDI and HIT-6 scores in our sample aligned with typical values seen in chronic pain and migraine populations.^40,41,42^

OLPs have demonstrated advantages for patients facing primary chronic pain, a condition characterized by the absence of actual or potential tissue damage, referred to as nociplastic pain.^14,43^ There is no evidence that OLP changes objective pathology. For example, previous trials have shown that OLPs do not impact objective outcomes, such as wound healing after punch biopsies or spinal motion in chronic back pain.^17,44^ Meta-analyses confirm their efficacy for subjective symptoms but not objective outcomes and highlight potential mechanisms of placebo effects (ie, expectation, conditioning, and bayesian signal processing).^14,21,45,46^ Migraine involves both peripheral and central pain mechanisms, particularly the trigeminovascular system, which leads to functional neuroplastic changes known as central sensitization.^47,48,49,50^ Migraine- and headache days and a 50% reduction thereof are well-established metrics for evaluating preventive migraine treatments.^3,23^ Although pain, such as migraine, is a subjective experience, our primary outcome measure of headache days leans toward objectivity as it ultimately hinges on a binary choice: whether a headache is present or not. Nonetheless, among our secondary, self-appraised outcomes, OLPs have been more extensively studied and shown to be efficient, particularly in addressing pain intensity, symptoms of depression, and fatigue.^21,45^

OLPs were well tolerated, with only 8 patients (14%) reporting adverse symptoms and a mean symptom score substantially lower than that typically observed with antidepressants used for migraine in the general population.^31^ Aside from possible ingredient intolerance, reported symptoms likely reflect misattributed interoceptive sensations, consistent with nocebo effects.^51,52,53,54^

The mechanisms underlying OLP efficacy remain unclear. In a qualitative study, Haas et al^55^ identified feelings of hope, uncertainty, and curiosity in placebo-treated patients with irritable bowel syndrome. Interestingly, their OLP-treated patients reported more self-examination, ambivalent feelings, and active engagement in their therapy compared with patients receiving deceptive placebos. This increased introspection might explain why participants in our trial noticed a more substantial positive change due to OLP.

According to ritual theory, placebo effects arise from healing rituals rooted in the patient-physician relationship.^56,57^ Substantial evidence indicates that the patient-physician relationship is crucial in enhancing placebo effects. In our trial, the minimal direct interaction, the use of prepackaged medication, and the standardized video explanations may have attenuated this relational element, potentially leading to an underestimation of OLP effects compared with previous studies.^16,58^

### Limitations

This study has limitations. First, it is inherent to the OLP approach that patients are not blinded to their treatment. However, we took specific measures to mitigate systematic errors, such as implementing an appropriate control group to address differential bias. Additionally, before randomization, all patients received the same information about the trial, including the OLP treatment, through a standardized video presentation. Second, COVID-19–related restrictions impeded recruitment, contributing to a reduced overall sample size and an imbalance across centers. Combined with a smaller-than-expected effect size of the primary end point, this may have resulted in insufficient power to detect between-group differences, limiting the interpretability of the null finding. Nevertheless, we accounted for site effects in our analyses, and with the exception of mental health (measured by the SF-12 MCS), our analyses did not reveal any center-specific biases. Although preregistration and false discovery rate correction were used to reduce bias, the secondary results should be interpreted with caution due to the nonsignificant primary outcome.^59^

## Conclusions

In this RCT, a 3-month OLP treatment combined with stable TAU did not reduce the number of headache or migraine days. However, OLP treatment was associated with relevant improvements in pain-related disability and quality of life, and patients reported a greater global impression of change after trial participation. These findings are consistent with previous research suggesting that OLPs may preferentially influence subjective, patient-reported outcomes rather than objective clinical measures. Although more research is needed, OLPs, as an adjunct to optimize reference treatment for migraine prevention, could potentially be a safe and suitable complementary option for patients with migraine, especially those who prefer nonpharmacologic approaches.

## Notes

Supplement 1.
Trial Protocol

Supplement 2. **eMethods.** Supplementary Methods **eFigure 1.** Study Design **eFigure 2.** Migraine Days **eFigure 3.** Mean Pain Intensity **eFigure 4.** Patient Global Impression of Change **eTable 1.** Generalized Linear Mixed-Effects Model – Monthly Headache Days **eTable 2.** Exploratory: Generalized Linear Mixed-Effects Model – Monthly Headache Days **eTable 3.** Generalized Linear Mixed-Effects Model – Monthly Migraine Days **eTable 4.** Exploratory: Generalized Linear Mixed-Effects Model – Monthly Migraine Days **eTable 5.** Robust Linear Mixed-Effects Model – Mean Pain Intensity **eTable 6.** Exploratory: Robust Linear Mixed-Effects Model – Mean Pain Intensity **eTable 7.** Generalized Linear Mixed-Effects Model – Rescue Medication Days **eTable 8.** Sensitivity: Hurdle Model – Rescue Medication Days **eTable 9.** Exploratory: Generalized Linear Mixed-Effects Model – Rescue Medication Days **eTable 10.** Exploratory/Sensitivity: Hurdle Model – Rescue Medication Days **eTable 11.** Robust Linear Mixed-Effects Model – SF-12 Mental Health **eTable 12.** Exploratory: Robust Linear Mixed-Effects Model – SF-12 Mental Health **eTable 13.** Robust Linear Mixed-Effects Model – SF-12 Physical Health **eTable 14.** Exploratory: Robust Linear Mixed-Effects Model – SF-12 Physical Health **eTable 15.** Robust Linear Mixed-Effects Model – Pain Disability Index **eTable 16.** Exploratory: Robust Linear Mixed-Effects Model – Pain Disability Index **eTable 17.** Robust Linear Mixed-Effects Model – Headache Impact Test 6 **eTable 18.** Exploratory: Robust Linear Mixed-Effects Model – Headache Impact Test 6

Supplement 3.
Data Sharing Statement
