# Supplementary material for: Open-Label Placebos as Adjunct for the Preventive Treatment of Migraine: A Randomized Clinical Trial
Source: JAMA Netw Open. 2025 Oct 8;8(10):e2535739. doi: 10.1001/jamanetworkopen.2025.35739 (PMC12509028; doi:10.1001/jamanetworkopen.2025.35739)
Supplement: Supplement 1. — Trial Protocol [file jamanetwopen-e2535739-s001.pdf]

Supplemental Online Content

Kleine-Borgmann J, Schmidt K, Ludwig L, et al. Open-label placebos as adjunct for the prevention treatment of migraine: a randomized clinical trial. *JAMA Netw Open*. 2025;8(10):e2535739. doi:10.1001/jamanetworkopen.2025.35739

eMethods. Supplementary Methods

eFigure 1. Study Design

eFigure 2. Migraine Days

eFigure 3. Mean Pain Intensity

eFigure 4. Patient Global Impression of Change

eTable 1. Generalized Linear Mixed-Effects Model – Monthly Headache Days

eTable 2. Exploratory: Generalized Linear Mixed-Effects Model – Monthly Headache Days

eTable 3. Generalized Linear Mixed-Effects Model – Monthly Migraine Days

eTable 4. Exploratory: Generalized Linear Mixed-Effects Model – Monthly Migraine Days

eTable 5. Robust Linear Mixed-Effects Model – Mean Pain Intensity

eTable 6. Exploratory: Robust Linear Mixed-Effects Model – Mean Pain Intensity

eTable 7. Generalized Linear Mixed-Effects Model – Rescue Medication Days

eTable 8. Sensitivity: Hurdle Model – Rescue Medication Days

eTable 9. Exploratory: Generalized Linear Mixed-Effects Model – Rescue Medication Days

eTable 10. Exploratory/Sensitivity: Hurdle Model – Rescue Medication Days

eTable 11. Robust Linear Mixed-Effects Model – SF-12 Mental Health

eTable 12. Exploratory: Robust Linear Mixed-Effects Model – SF-12 Mental Health

eTable 13. Robust Linear Mixed-Effects Model – SF-12 Physical Health

eTable 14. Exploratory: Robust Linear Mixed-Effects Model – SF-12 Physical Health

eTable 15. Robust Linear Mixed-Effects Model – Pain Disability Index

eTable 16. Exploratory: Robust Linear Mixed-Effects Model – Pain Disability Index

eTable 17. Robust Linear Mixed-Effects Model – Headache Impact Test 6

eTable 18. Exploratory: Robust Linear Mixed-Effects Model – Headache Impact Test 6

This supplemental material has been provided by the authors to give readers additional information about their work.

eMethods. Supplementary Methods

1.1 Study protocol

For study protocol please see original protocol publication:

Schmidt, K., Kleine-Borgmann, J., Holle-Lee, D., Gaul, C., & Bingel, U. (2021). Impact of a 12-week open-label placebo treatment on headache days in episodic and chronic migraine: a study protocol for a parallel-group, multicentre, randomised controlled trial. BMJ Open, 11(6), e045969. https://doi.org/10.1136/bmjopen-2020-045969

1.2 Patient instructions

1.2.1 German original of the instruction video shown to the patients.

Author U.B.: Liebe Patientin und lieber Patient,

Ich freue mich, dass Sie an dieser Studie teilnehmen. In der Universitätsmedizin Essen bieten wir Patienten mit Migräne ein fachübergreifendes Behandlungskonzept und vor allem gehen wir dem Thema Schmerz auch wissenschaftlich auf den Grund.

Erste internationale Studien legen nahe, dass sowohl akute als auch chronische Schmerzen durch eine Behandlung mit Placebotabletten deutlich gelindert werden können. Diese spannenden Befunde haben auch Einzug in die Medien gehalten.

Der folgende Beitrag eines amerikanischen Nachrichtensenders fasst diese sehr schön zusammen:

[American News Outlet, see below]

Author U.B.: Wie dieser Zusammenschnitt zeigt, scheint Palcebobehandlung bei verschiedenen Schmerzsyndromen tatsächlich wirken zu können. Diesem Phänomen möchten wir in der aktuellen Studie nachgehen. Insbesondere möchten wir neben den Einflüssen auf den Kopfschmerz an sich, auch den Einfluss der Placebobehandlung auf ihr Befinden, also ihre Alltagseinschränkung durch die Migräne untersuchen. Außerdem hoffen wir durch die begleitenden experimentellen Untersuchungen, wie beispielsweise die MRT-Messung, besser zu verstehen, wer von einer solchen Therapie am besten profitiert und welche Mechanismen der möglichen Wirkung der Placebotabletten zugrunde liegen. Ich danke Ihnen sehr für Ihre Teilnahme.

1.2.2 English translation of the instruction video shown to the patients.

Author U.B.: Dear patient,

I am pleased that you are taking part in this study. At University Medicine Essen, we offer comprehensive multidisciplinary medical care to patients with migraine but also aim to understand pain through scientific research.

First, international studies suggest that both acute and chronic pain can be reduced significantly when treated with placebo tablets. These interesting findings have also found their way into the media.

© 2025 Kleine-Borgmann J et al. *JAMA Network Open.*

The following article by an American news channel provides a nice summary of the findings.

[American News Outlet begins]

Speaker: Can the knowledge of taking a placebo actually improve your health? Studies show that it is possible. Some patients may no longer need proper medication. More and more patients are prescribed placebos.

Speaker: It looks like a normal pill but turns out to be a placebo. These, doctors confirm, can be used to treat some of the most common diseases.

Patient: I felt fantastic, better than ever.

Speaker: For Linda Buonanno, the placebo pills worked. She suffers from irritable bowel syndrome, which often develops without warning.

Patient: I felt terrible, I had no life. I couldn't plan or do anything.

Speaker: When she found out about the study, she applied immediately.

Patient: I was very happy.

Speaker: But she was shocked when she heard that she was getting a placebo instead of real medication. We said: You don't have to believe it, just do it. Even if it's kind of a crazy idea.

Patient: I was so disappointed, I said: A placebo? A sugar pill, is that a joke? It's never going to work.

Speaker: But it worked. Her symptoms disappeared.

Patient: I'm making plans again, I don't have to worry anymore. I live my life again.

Speaker: In another study the same was done with migraine patients. Their pain was reduced by 30 percent.

Expert 1: This is an incredible thing.

Speaker: Psychologist Dr Stratyner says other factors may play a role.

Expert 2: I think the patients might think: Hm, I am wondering if it really is a placebo. Maybe I'm just being told that.

Speaker: Kaptchuk says that there are physiological reasons why placebos activate the same neurotransmitters as many powerful drugs.

Expert 1: We have our own pharmacy for certain diseases.

Speaker: But there are limits to the effect of placebo.

Expert 1: We will not be able to shrink a tumor with the placebo pill.

Speaker: But for certain diseases, placebos could fundamentally change the treatment.

Expert 1: If a placebo helps, this would be the best approach instead of putting patients on strong medication for a long time.

© 2025 Kleine-Borgmann J et al. *JAMA Network Open.*

[News outlet ends]

Author U.B.: As this summary shows, placebo treatment may indeed have a positive effect on various chronic pain syndromes. We would like to investigate this phenomenon in the current study. In addition to the effect on headache itself, we would also like to examine the influence on your well-being, which is your functional disability due to migraine. Furthermore, by accompanying experimental methods, such as magnetic resonance imaging, we hope to better understand who benefits most from such a therapy and which mechanisms underlie the potential effects of placebo tablets. Thank you very much for your participation.

© 2025 Kleine-Borgmann J et al. *JAMA Network Open.*

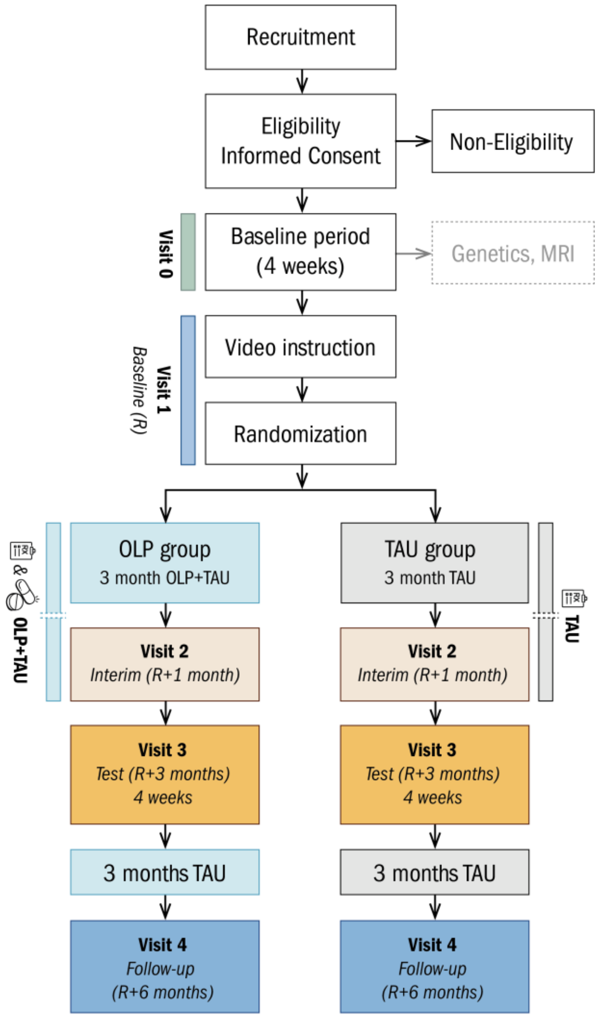

eFigure 1. Study design.

The figure outlines the pre-registered study design based on the study protocol see [24], featuring a treatment phase of either three months of OLP in addition to TAU or TAU alone. Genetics and MRI data are not included in this manuscript and will be detailed in a separate report. *OLP, Open-label placebo; TAU, treatment as usual; R, Randomization.*

116

© 2025 Kleine-Borgmann J et al. *JAMA Network Open.*

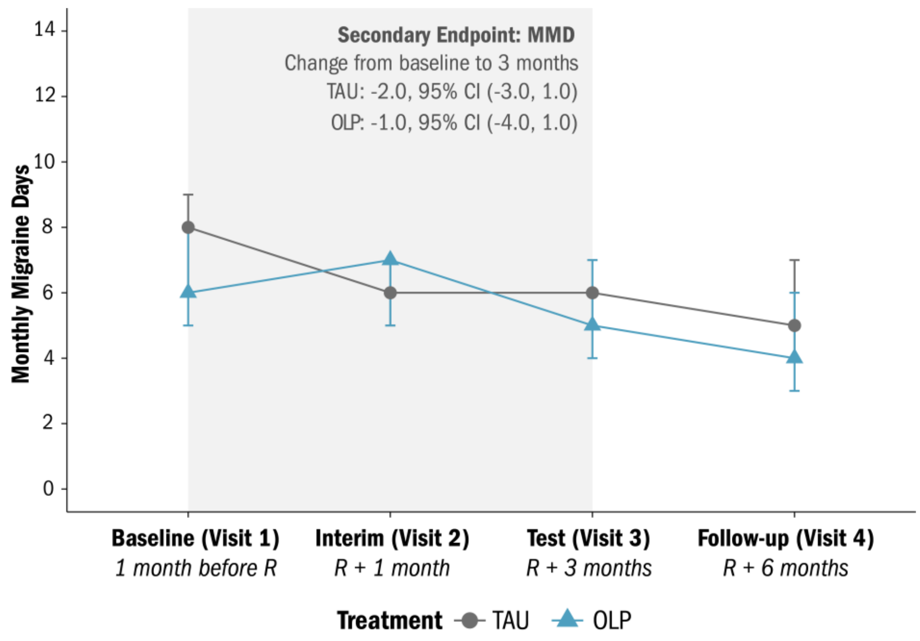

eFigure 2. Migraine days.

Median migraine days (secondary end point) in the past 4-week period and bootstrapped 95% confidence intervals are shown per group. *R, Randomization; TAU, treatment as usual group; OLP, Open-label placebo group.*

117

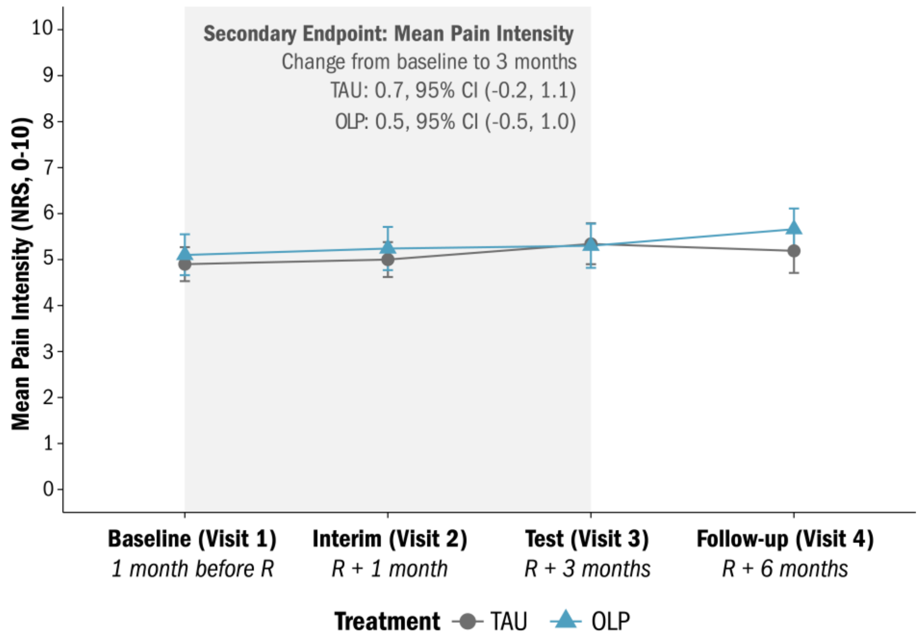

eFigure 3. Mean pain intensity.

The figure presents the mean pain intensity with bootstrapped 95% confidence intervals in the past 4-week period per group. *R, Randomization; TAU, treatment as usual group; OLP, Open-label placebo group.*

118

© 2025 Kleine-Borgmann J et al. *JAMA Network Open.*

© 2025 Kleine-Borgmann J et al. *JAMA Network Open.*

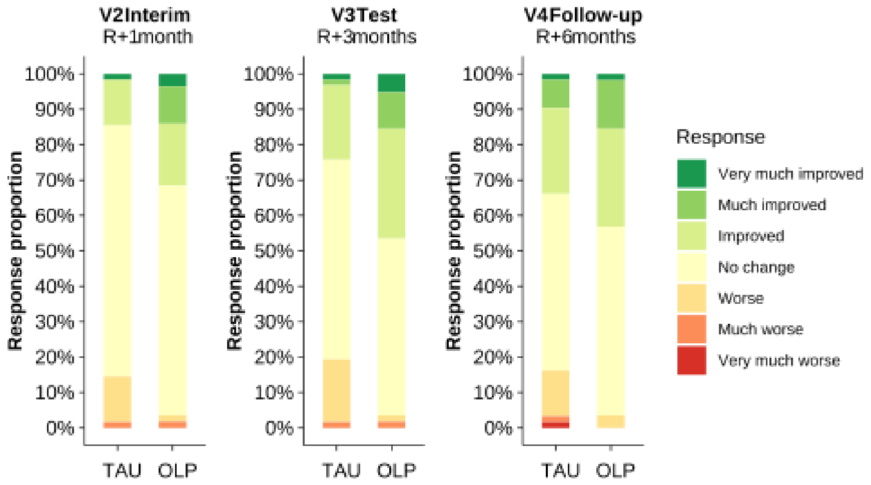

eFigure 4. Patient Global Impression of Change.

The figure illustrates the item proportions for the OLP and TAU group separately at visit 2 (V2), visit 3 (V3), and visit 4 (V4). *TAU, treatment as usual group; OLP, Open-label placebo group.*

120

© 2025 Kleine-Borgmann J et al. *JAMA Network Open.*

1.3 eTables

1.3.1 Monthly Headache Days

eTable 1. Generalized Linear Mixed-Effects Model – Monthly Headache Days

Fixed effects	Incidence Rate Ratios	95% CI	*P*	P_{adj}
(Intercept)	7.90	6.69 – 9.32	**<.001**	**<.001**
Group: OLP vs. TAU	0.88	0.70 – 1.11	.284	.356
Time: Visit 3 vs. visit 1	0.90	0.80 – 1.02	.097	.243
Center: Frankfurt vs. Essen	1.06	0.78 – 1.43	.713	.713
Interaction: OLP x visit 3	0.89	0.75 – 1.07	.206	.343

Results of a generalized linear mixed-effects model following a Poisson distribution including patient ID as random effect (σ^2 = 0.12, N = 120). Only data from visit 1 and visit 3 were included in the model. Marginal R^2 = 0.04, conditional R^2 = 0.72, AIC = 1370.59. Residual diagnostics indicated that the model fit the data well. Simulated residuals showed no evidence of non-uniformity (*P*=.93), overdispersion (*P*=.86), or zero-inflation (*P*=.06). Multicollinearity was low among fixed effects (VIF<2). P values were adjusted for multiple comparisons by False Discovery Rate (FDR) correction. *OLP: Open-Label Placebo, TAU: Treatment as usual.*

eTable 2. Exploratory: Generalized Linear Mixed-Effects Model – Monthly Headache Days

Fixed effects	Incidence Rate Ratios	95% CI	*P*
(Intercept)	7.72	6.50 – 9.19	**<.001**
Group: OLP vs. TAU	0.88	0.69 – 1.13	.315
Time: Visit 2 vs. visit 1	0.89	0.79 – 1.01	.061
Time: Visit 3 vs. visit 1	0.90	0.80 – 1.02	.098
Time: Visit 4 vs. visit 1	0.84	0.74 – 0.95	**.005**
Center: Frankfurt vs. Essen	1.08	0.80 – 1.47	.615
Interaction: OLP x visit 2	0.99	0.83 – 1.19	.942
Interaction: OLP x visit 3	0.89	0.75 – 1.07	.208
Interaction: OLP x visit 4	0.90	0.74 – 1.08	.258

Results of a generalized linear mixed-effects model following a Poisson distribution including patient ID as random effect (σ^2 = 0.12, N = 120). Data from visit 1 to 4 were included in the model. Marginal R^2 = 0.03, conditional R^2 = 0.74, AIC = 2503.69. Residual diagnostics indicated that the model fit the data well. Simulated residuals showed no evidence of non-uniformity (*P*=.65) or overdispersion (*P*=.98) but moderate probability of zero-inflation (*P*=.02). Multicollinearity was low to moderate among fixed effects (VIF<6.5). *OLP: Open-Label Placebo, TAU: Treatment as usual.*

© 2025 Kleine-Borgmann J et al. *JAMA Network Open.*

1.3.2 Monthly Migraine Days

**eTable 3. Generalized Linear Mixed-Effects Model – Monthly Migraine Days**				
**Fixed effects**	**Incidence Rate Ratios**	**95% CI**	***P***	***P*$_{adj}$**
(Intercept)	7.05	6.01 – 8.28	**<.001**	**<.001**
Group: OLP vs. TAU	0.90	0.72 – 1.13	.377	.463
Time: Visit 3 vs. visit 1	0.88	0.77 – 0.99	**.041**	.103
Center: Frankfurt vs. Essen	1.15	0.86 – 1.52	.350	.463
Interaction: OLP x visit 3	0.93	0.77 – 1.12	.463	.463

Results of a generalized linear mixed-effects model following a Poisson distribution including patient ID as random effect (σ^2 = 0.13, N = 120). Only data from visit 1 and visit 3 were included in the model. Marginal R^2 = 0.04, conditional R^2 = 0.67, AIC = 1331.78. Residual diagnostics indicated that the model fit the data well. Simulated residuals showed no evidence of non-uniformity (*P*=.98), overdispersion (*P*=.66), or zero-inflation (*P*=.20). Multicollinearity was low among fixed effects (VIF≤2). P values were adjusted for multiple comparisons by False Discovery Rate (FDR) correction. *OLP: Open-Label Placebo, TAU: Treatment as usual.*

**eTable 4. Exploratory: Generalized Linear Mixed-Effects Model – Monthly Migraine Days**			
**Fixed effects**	**Incidence Rate Ratios**	**95% CI**	***P***
(Intercept)	6.92	5.88 – 8.14	**<.001**
Group: OLP vs. TAU	0.92	0.73 – 1.15	.467
Time: Visit 2 vs. visit 1	0.87	0.77 – 0.99	**.033**
Time: Visit 3 vs. visit 1	0.88	0.77 – 1.00	**.042**
Time: Visit 4 vs. visit 1	0.82	0.71 – 0.93	**.002**
Center: Frankfurt vs. Essen	1.17	0.89 – 1.55	.266
Interaction: OLP x visit 2	1.06	0.88 – 1.28	.540
Interaction: OLP x visit 3	0.93	0.77 – 1.13	.465
Interaction: OLP x visit 4	0.90	0.74 – 1.10	.300

Results of a generalized linear mixed-effects model following a Poisson distribution including patient ID as random effect (σ^2 = 0.14, N = 120). Data from visit 1 to 4 were included in the model. Marginal R^2 = 0.04, conditional R^2 = 0.68, AIC = 2452.29. Residual diagnostics indicated that the model fit the data well. Simulated residuals showed no evidence of non-uniformity (*P*=.99), overdispersion (*P*=.65), or zero-inflation (*P*=.23). Multicollinearity was low to moderate among fixed effects (VIF≤7). *OLP: Open-Label Placebo, TAU: Treatment as usual.*

© 2025 Kleine-Borgmann J et al. *JAMA Network Open.*

130 1.3.3 Mean Pain Intensity

eTable 5. Robust Linear Mixed-Effects Model – Mean Pain Intensity				
**Fixed effects**	**Estimates**	**95% CI**	***P***	***P*adj**
(Intercept)	4.93	4.50 – 5.36	**<.001**	**<.001**
Group: OLP vs. TAU	0.11	-0.48 – 0.71	.712	.712
Time: Visit 3 vs. visit 1	0.43	0.14 – 0.71	**.004**	**.009**
Center: Frankfurt vs. Essen	-0.19	-0.98 – 0.59	.626	.712
Interaction: OLP x visit 3	-0.17	-0.58 – 0.24	.418	.697

Results of a robust linear mixed-effects model including patient ID as random effect (σ^2 = 0.62, N = 120). Only data from visit 1 and visit 3 were included in the model. Marginal R^2 = 0.01, conditional R^2 = 0.77, RMSE = 0.682. Residual diagnostics indicated approximate linearity and constant variance. A QQ plot suggested moderate deviation from normality, which is expected in robust modeling. P values were adjusted for multiple comparisons by False Discovery Rate (FDR) correction. *OLP: Open-Label Placebo, TAU: Treatment as usual.*

131

eTable 6. Exploratory: Robust Linear Mixed-Effects Model – Mean Pain Intensity			
**Fixed effects**	**Estimates**	**95% CI**	***P***
(Intercept)	4.97	4.54 – 5.40	**<.001**
Group: OLP vs. TAU	0.13	-0.46 – 0.73	.661
Time: Visit 2 vs. visit 1	0.15	-0.13 – 0.44	.293
Time: Visit 3 vs. visit 1	0.43	0.15 – 0.72	**.003**
Time: Visit 4 vs. visit 1	0.35	0.06 – 0.64	**.017**
Center: Frankfurt vs. Essen	-0.32	-1.08 – 0.44	.415
Interaction: OLP x visit 2	0.02	-0.39 – 0.43	.934
Interaction: OLP x visit 3	-0.21	-0.62 – 0.20	.312
Interaction: OLP x visit 4	0.08	-0.34 – 0.50	.719

Results of a robust linear mixed-effects model including patient ID as random effect (σ^2 = 0.62, N = 120). Data from visit 1 to 4 were included in the model. Marginal R^2 = 0.02, conditional R^2 = 0.77, RMSE = 0.827. Residual diagnostics indicated approximate linearity and constant variance. A QQ plot suggested moderate deviation from normality, which is expected in robust modeling. *OLP: Open-Label Placebo, TAU: Treatment as usual.*

132

133

© 2025 Kleine-Borgmann J et al. *JAMA Network Open.*

1.3.4 Rescue Medication Days

eTable 7. Generalized Linear Mixed-Effects Model – Rescue Medication Days

Fixed effects	Incidence Rate Ratios	95% CI	*P*	P_{adj}
(Intercept)	5.09	4.20 – 6.19	**<.001**	**<.001**
Group: OLP vs. TAU	0.83	0.63 – 1.08	.169	.282
Time: Visit 3 vs. visit 1	0.96	0.83 – 1.11	.575	.575
Center: Frankfurt vs. Essen	1.20	0.85 – 1.69	.311	.388
Interaction: OLP x visit 3	0.83	0.67 – 1.04	.099	.248

Results of a generalized linear mixed-effects model following a Poisson distribution including patient ID as random effect (σ^2 = 0.17, N = 120). Only data from visit 1 and visit 3 were included in the model. Marginal R^2 = 0.05, conditional R^2 = 0.71, AIC = 1241.12. Residual diagnostics indicated that the model fit the data well. Simulated residuals showed no evidence of non-uniformity (*P*=.54) or overdispersion (*P*=.24). The zero-inflation test via comparison to expected zeros with simulation under H0 = fitted model revealed a high probability of zero-inflation (*P*<.001). Multicollinearity was low among fixed effects (VIF<2). P values were adjusted for multiple comparisons by False Discovery Rate (FDR) correction. *OLP: Open-Label Placebo, TAU: Treatment as usual.*

eTable 8. Sensitivity: Hurdle Model – Rescue Medication Days

Fixed effects	Incidence Rate Ratios	95% CI	*P*	P_{adj}
(Intercept)	5.78	4.90 – 6.81	**<.001**	**<.001**
Group: OLP vs. TAU	0.88	0.70 – 1.11	.880	.413
Time: Visit 3 vs. visit 1	0.95	0.82 – 1.10	.950	.543
Center: Frankfurt vs. Essen	1.07	0.81 – 1.41	.654	.654
Interaction: OLP x visit 3	0.88	0.70 – 1.11	.279	.413

Results of a Hurdle model following a truncated Poisson distribution including patient ID as random effect (σ^2 = 0.17, N = 120). Only data from visit 1 and visit 3 were included in the model. Marginal R^2 = 0.04, conditional R^2 = 0.56, AIC = 1251.22. The zero-inflated model included the group as fixed effect (IRR=2.48, 95% CI=0.97 to 6.33, *P*=0.057, P_{FDR}=0.133). P values were adjusted for multiple comparisons by False Discovery Rate (FDR) correction. *OLP: Open-Label Placebo, TAU: Treatment as usual.*

**eTable 9. Exploratory: Generalized Linear Mixed-Effects Model – Rescue Medication Days**			
**Fixed effects**	**Incidence Rate Ratios**	**95% CI**	***P***
(Intercept)	4.97	4.04 – 6.12	**<.001**
Group: OLP vs. TAU	0.82	0.61 – 1.10	.182
Time: Visit 2 vs. visit 1	0.91	0.78 – 1.05	.186
Time: Visit 3 vs. visit 1	0.96	0.83 – 1.11	.576
Time: Visit 4 vs. visit 1	0.89	0.77 – 1.03	.128
Center: Frankfurt vs. Essen	1.20	0.84 – 1.73	.322
Interaction: OLP x visit 2	0.96	0.77 – 1.19	.713
Interaction: OLP x visit 3	0.83	0.67 – 1.04	.100
Interaction: OLP x visit 4	0.83	0.66 – 1.05	.124

Results of a generalized linear mixed-effects model following a Poisson distribution including patient ID as random effect (σ^2 = 0.17, N = 120). Data from visit 1 to 4 were included in the model. Marginal R^2 = 0.05, conditional R^2 = 0.75, AIC = 2277.16. Residual diagnostics indicated that the model fit the data well. Simulated residuals showed no evidence of non-uniformity (*P*=.07) but overdispersion (*P*=.02) and zero-inflation (*P*<.001). Multicollinearity was low to moderate among fixed effects (VIF<6). *OLP: Open-Label Placebo, TAU: Treatment as usual.*

**eTable 10. Exploratory/Sensitivity: Hurdle Model – Rescue Medication Days**			
**Fixed effects**	**Incidence Rate Ratios**	**95% CI**	***P***
(Intercept)	5.70	4.83 – 6.73	**<.001**
Group: OLP vs. TAU	0.89	0.70 – 1.13	.341
Time: Visit 2 vs. visit 1	0.91	0.78 – 1.05	.205
Time: Visit 3 vs. visit 1	0.95	0.82 – 1.10	.463
Time: Visit 4 vs. visit 1	0.89	0.76 – 1.04	.129
Center: Frankfurt vs. Essen	1.08	0.82 – 1.41	.582
Interaction: OLP x visit 2	0.96	0.77 – 1.20	.713
Interaction: OLP x visit 3	0.87	0.69 – 1.09	.234
Interaction: OLP x visit 4	0.93	0.73 – 1.18	.539

Results of a Hurdle model following a truncated Poisson distribution including patient ID as random effect (σ^2 = 0.18, N = 120). Data from visit 1 to 4 were included in the model. Marginal R^2 = 0.03, conditional R^2 = 0.57, AIC = 2324.60. The zero-inflated model included the group as fixed effect (IRR=2.32, 95% CI=1.25 to 4.29, *P*=.008). *OLP: Open-Label Placebo, TAU: Treatment as usual.*

© 2025 Kleine-Borgmann J et al. *JAMA Network Open.*

1.3.5 Quality of Life: SF-12 Mental Health Scores

eTable 11. Robust Linear Mixed-Effects Model – SF-12 Mental Health				
**Fixed effects**	**Estimates**	**95% CI**	***P***	***P*_adj**
(Intercept)	47.75	45.26 – 50.24	**<.001**	**<.001**
Group: OLP vs. TAU	0.58	-2.88 – 4.04	.741	.741
Time: Visit 3 vs. visit 1	-1.14	-3.20 – 0.92	.275	.344
Center: Frankfurt vs. Essen	-5.03	-9.40 – -0.65	**.025**	.062
Interaction: OLP x visit 3	2.72	-0.24 – 5.68	.072	.120

Results of a robust linear mixed-effects model including patient ID as random effect (σ^2 = 32.15, N = 120). Only data from visit 1 and visit 3 were included in the model. Marginal R^2 = 0.05, conditional R^2 = 0.65, RMSE = 4.56. Residual diagnostics indicated approximate linearity and constant variance. A QQ plot suggested moderate deviation from normality, which is expected in robust modeling. P values were adjusted for multiple comparisons by False Discovery Rate (FDR) correction. *OLP: Open-Label Placebo, TAU: Treatment as usual.*

eTable 12. Exploratory: Robust Linear Mixed-Effects Model – SF-12 Mental Health			
**Fixed effects**	**Estimates**	**95% CI**	***P***
(Intercept)	47.78	45.30 – 50.25	**<.001**
Group: OLP vs. TAU	0.44	-3.02 – 3.89	.803
Time: Visit 2 vs. visit 1	-0.11	-2.11 – 1.88	.913
Time: Visit 3 vs. visit 1	-1.18	-3.17 – 0.82	.247
Time: Visit 4 vs. visit 1	-1.18	-3.17 – 0.82	.247
Center: Frankfurt vs. Essen	-4.45	-8.62 – -0.29	**.036**
Interaction: OLP x visit 2	-0.12	-2.99 – 2.76	.936
Interaction: OLP x visit 3	2.97	0.10 – 5.84	**.042**
Interaction: OLP x visit 4	1.61	-1.26 – 4.48	.270

Results of a robust linear mixed-effects model including patient ID as random effect (σ^2 = 30.37, N = 120). Data from visit 1 to 4 were included in the model. Marginal R^2 = 0.04, conditional R^2 = 0.67, RMSE = 5.46. Residual diagnostics indicated approximate linearity and constant variance. A QQ plot suggested moderate deviation from normality, which is expected in robust modeling. *OLP: Open-Label Placebo, TAU: Treatment as usual.*

© 2025 Kleine-Borgmann J et al. *JAMA Network Open.*

1.3.6 Quality of Life: SF-12 Physical Health Scores

eTable 13. Robust Linear Mixed-Effects Model – SF-12 Physical Health				
**Fixed effects**	**Estimates**	**95% CI**	***P***	***P_{adj}***
(Intercept)	39.14	36.82 – 41.45	**<.001**	**<.001**
Group: OLP vs. TAU	-0.99	-4.22 – 2.23	.544	.645
Time: Visit 3 vs. visit 1	0.47	-1.55 – 2.50	.645	.645
Center: Frankfurt vs. Essen	-2.14	-6.17 – 1.89	.296	.493
Interaction: OLP x visit 3	4.25	1.33 – 7.17	**.004**	**.011**

Results of a robust linear mixed-effects model including patient ID as random effect (σ^2 = 31.23, N = 120). Only data from visit 1 and visit 3 were included in the model. Marginal R^2 = 0.05, conditional R^2 = 0.61, RMSE = 4.35. Residual diagnostics indicated approximate linearity and constant variance. A QQ plot suggested moderate deviation from normality, which is expected in robust modeling. P values were adjusted for multiple comparisons by False Discovery Rate (FDR) correction. *OLP: Open-Label Placebo, TAU: Treatment as usual.*

eTable 14. Exploratory: Robust Linear Mixed-Effects Model – SF-12 Physical Health			
**Fixed effects**	**Estimates**	**95% CI**	***P***
(Intercept)	38.99	36.69 – 41.28	**<.001**
Group: OLP vs. TAU	-0.87	-4.08 – 2.33	.593
Time: Visit 2 vs. visit 1	0.76	-1.17 – 2.70	.438
Time: Visit 3 vs. visit 1	0.46	-1.47 – 2.40	.637
Time: Visit 4 vs. visit 1	1.03	-0.90 – 2.96	.295
Center: Frankfurt vs. Essen	-1.53	-5.33 – 2.27	.430
Interaction: OLP x visit 2	2.71	-0.07 – 5.50	.056
Interaction: OLP x visit 3	4.11	1.33 – 6.89	**.004**
Interaction: OLP x visit 4	3.37	0.59 – 6.15	**.018**

Results of a robust linear mixed-effects model including patient ID as random effect (σ^2 = 28.48, N = 120). Data from visit 1 to 4 were included in the model. Marginal R^2 = 0.04, conditional R^2 = 0.64, RMSE = 4.74. Residual diagnostics indicated approximate linearity and constant variance. A QQ plot suggested moderate deviation from normality, which is expected in robust modeling. *OLP: Open-Label Placebo, TAU: Treatment as usual.*

© 2025 Kleine-Borgmann J et al. *JAMA Network Open.*

1.3.7 Disability: PDI Scores

eTable 15. Robust Linear Mixed-Effects Model – Pain Disability Index				
**Fixed effects**	**Estimates**	**95% CI**	***P***	P_{adj}
(Intercept)	20.61	17.60 – 23.62	**<.001**	**<.001**
Group: OLP vs. TAU	-0.12	-4.30 – 4.06	.956	.956
Time: Visit 3 vs. visit 1	0.82	-1.31 – 2.94	.449	.561
Center: Frankfurt vs. Essen	4.94	-0.52 – 10.41	.076	.127
Interaction: OLP x visit 3	-5.96	-9.01 – -2.92	**<.001**	**<.001**

Results of a robust linear mixed-effects model including patient ID as random effect (σ^2 = 33.72, N = 120). Only data from visit 1 and visit 3 were included in the model. Marginal R^2 = 0.06, conditional R^2 = 0.75, RMSE = 5.54. Residual diagnostics indicated approximate linearity and constant variance. A QQ plot suggested moderate deviation from normality, which is expected in robust modeling. P values were adjusted for multiple comparisons by False Discovery Rate (FDR) correction. *OLP: Open-Label Placebo, TAU: Treatment as usual.*

eTable 16. Exploratory: Robust Linear Mixed-Effects Model – Pain Disability Index			
**Fixed effects**	**Estimates**	**95% CI**	***P***
(Intercept)	20.96	17.76 – 24.16	**<.001**
Group: OLP vs. TAU	-0.45	-4.90 – 3.99	.842
Time: Visit 2 vs. visit 1	1.14	-0.92 – 3.21	.278
Time: Visit 3 vs. visit 1	0.88	-1.20 – 2.95	.408
Time: Visit 4 vs. visit 1	-0.62	-2.70 – 1.46	.556
Center: Frankfurt vs. Essen	4.75	-0.93 – 10.43	.101
Interaction: OLP x visit 2	-3.86	-6.84 – -0.88	**.011**
Interaction: OLP x visit 3	-5.98	-8.96 – -3.00	**<.001**
Interaction: OLP x visit 4	-4.40	-7.38 – -1.41	**.004**

Results of a robust linear mixed-effects model including patient ID as random effect (σ^2 = 32.59, N = 120). Data from visit 1 to 4 were included in the model. Marginal R^2 = 0.06, conditional R^2 = 0.79, RMSE = 5.89. Residual diagnostics indicated approximate linearity and constant variance. A QQ plot suggested moderate deviation from normality, which is expected in robust modeling. *OLP: Open-Label Placebo, TAU: Treatment as usual.*

© 2025 Kleine-Borgmann J et al. *JAMA Network Open.*

1.3.8 Disability: HIT-6

eTable 17. Robust Linear Mixed-Effects Model – Headache Impact Test 6				
**Fixed effects**	**Estimates**	**95% CI**	***P***	***P*$_{adj}$**
(Intercept)	64.39	62.95 – 65.82	**<.001**	**<.001**
Group: OLP vs. TAU	-0.67	-2.66 – 1.32	.507	.790
Time: Visit 3 vs. visit 1	-0.10	-1.08 – 0.87	.834	.834
Center: Frankfurt vs. Essen	-0.63	-3.24 – 1.97	.632	.790
Interaction: OLP x visit 3	-1.88	-3.28 – -0.48	**.009**	**.022**

Results of a robust linear mixed-effects model including patient ID as random effect (σ^2 = 7.22, N = 120). Only data from visit 1 and visit 3 were included in the model. Marginal R^2 = 0.04, conditional R^2 = 0.76, RMSE = 2.81. Residual diagnostics indicated approximate linearity and constant variance. A QQ plot suggested moderate deviation from normality, which is expected in robust modeling. P values were adjusted for multiple comparisons by False Discovery Rate (FDR) correction. *OLP: Open-Label Placebo, TAU: Treatment as usual.*

eTable 18. Exploratory: Robust Linear Mixed-Effects Model – Headache Impact Test 6			
**Fixed effects**	**Estimates**	**95% CI**	***P***
(Intercept)	64.28	62.73 – 65.83	**<.001**
Group: OLP vs. TAU	-1.04	-3.19 – 1.12	.344
Time: Visit 2 vs. visit 1	-0.76	-1.77 – 0.25	.141
Time: Visit 3 vs. visit 1	-0.04	-1.05 – 0.98	.944
Time: Visit 4 vs. visit 1	-1.22	-2.23 – -0.21	**.018**
Center: Frankfurt vs. Essen	-0.03	-2.77 – 2.71	.983
Interaction: OLP x visit 2	-0.78	-2.24 – 0.68	.295
Interaction: OLP x visit 3	-1.99	-3.45 – -0.53	**.008**
Interaction: OLP x visit 4	-0.34	-1.80 – 1.12	.650

Results of a robust linear mixed-effects model including patient ID as random effect (σ^2 = 7.84, N = 120). Data from visit 1 to 4 were included in the model. Marginal R^2 = 0.04, conditional R^2 = 0.78, RMSE = 2.94. Residual diagnostics indicated approximate linearity and constant variance. A QQ plot suggested moderate deviation from normality, which is expected in robust modeling. *OLP: Open-Label Placebo, TAU: Treatment as usual.*

© 2025 Kleine-Borgmann J et al. *JAMA Network Open.*
