# Supplementary material for: Open-Label Placebos as Adjunct for the Preventive Treatment of Migraine: A Randomized Clinical Trial
Source: JAMA Netw Open. 2025 Oct 8;8(10):e2535739. doi: 10.1001/jamanetworkopen.2025.35739 (PMC12509028; doi:10.1001/jamanetworkopen.2025.35739)
Supplement: Supplement 2. — eMethods. Supplementary Methods eFigure 1. Study Design eFigure 2. Migraine Days eFigure 3. Mean Pain Intensity eFigure 4. Patient Global Impression of Change eTable 1. Generalized Linear Mixed-Effects Model – Monthly Headache Days eTable 2. Exploratory: Generalized Linear Mixed-Effects Model – Monthly Headache Days eTable 3. Generalized Linear Mixed-Effects Model – Monthly Migraine Days eTable 4. Exploratory: Generalized Linear Mixed-Effects Model – Monthly Migraine Days eTable 5. Robust Linear Mixed-Effects Model – Mean Pain Intensity eTable 6. Exploratory: Robust Linear Mixed-Effects Model – Mean Pain Intensity eTable 7. Generalized Linear Mixed-Effects Model – Rescue Medication Days eTable 8. Sensitivity: Hurdle Model – Rescue Medication Days eTable 9. Exploratory: Generalized Linear Mixed-Effects Model – Rescue Medication Days eTable 10. Exploratory/Sensitivity: Hurdle Model – Rescue Medication Days eTable 11. Robust Linear Mixed-Effects Model – SF-12 Mental Health eTable 12. Exploratory: Robust Linear Mixed-Effects Model – SF-12 Mental Health eTable 13. Robust Linear Mixed-Effects Model – SF-12 Physical Health eTable 14. Exploratory: Robust Linear Mixed-Effects Model – SF-12 Physical Health eTable 15. Robust Linear Mixed-Effects Model – Pain Disability Index eTable 16. Exploratory: Robust Linear Mixed-Effects Model – Pain Disability Index eTable 17. Robust Linear Mixed-Effects Model – Headache Impact Test 6 eTable 18. Exploratory: Robust Linear Mixed-Effects Model – Headache Impact Test 6 [file jamanetwopen-e2535739-s002.pdf]

The impact of a 12-week open-label placebo treatment on headache days in episodic and chronic migraine

(Acronym: OLPIMI)

Study Protocol

Version 2.1 - October 13, 2020

This document was translated from German to English on May 5, 2025.

Investigator Initiated Trial
Prof. Dr. Ulrike Bingel
University Medicine Essen
Hufelandstrasse 55
45147 Essen
Germany
Phone: +49 0201 723 2446
E-Mail: Ulrike.Bingel@uk-essen.de

Table of Contents

1 Introduction 4

1.1 Scientific Background 4

2 Study Design and Objectives 7

2.1 Study Design 7

2.2 Objectives 7

2.2.1 Primary Objective 7

2.2.2 Secondary Objectives 8

2.2.3 Exploratory Objectives 8

3 Study population 9

3.1 Inclusion Criteria 9

3.2 Exclusion Criteria 9

4 Study Intervention 10

4.1 Study Treatment and Arms 10

4.2 Randomization 11

5 Endpoints and Assessments 12

5.1 Visit Schedule 12

5.2 Primary Endpoint 15

5.2.1 Definitions 15

5.3 Secondary Endpoints 16

5.4 Exploratory Endpoints 16

6 Statistical Considerations 18

6.1 Sample Size Calculation 18

6.2 Statistical Analysis Plan 18

7 References 20

1 Introduction

1.1 Scientific Background

With an estimated 1-year prevalence of around 12% (5.6% in men, 17.1% in women), migraine is the most common neurological disorder. As one of the ten leading causes of years lived with disability (YLD) worldwide, migraine is highly relevant not only on an individual level but also socioeconomically (James et al., 2018; Lipton et al., 2007). As a primary headache disorder, its diagnosis and classification are based on the criteria of the International Classification of Headache Disorders, 3rd edition (ICHD-III) (Olesen, 2018). The threshold distinguishing episodic migraine (EM) from chronic migraine (CM) is 15 headache days per month over the past three months.

While acute migraine attacks are often successfully treated with nonsteroidal anti-inflammatory drugs (NSAIDs) or triptans, prophylactic treatment—especially for CM—remains challenging and is frequently discontinued due to side effects that often occur before the onset of therapeutic benefit (Gallagher and Kunkel, 2003). Additionally, due to economic constraints and limited resources, newer specific pharmacological treatments (e.g., CGRP and CGRP receptor antibodies, onabotulinumtoxinA) and non-pharmacological approaches (e.g., inpatient multimodal therapies) are only accessible to a subset of patients (Negro and Martelletti, 2019; Sussman et al., 2018).

Recently, randomized controlled trials (RCTs) have demonstrated that open-label placebo (OLP) treatments can lead to significant and clinically relevant symptom improvements in chronic pain conditions (e.g., chronic low back pain, irritable bowel syndrome) as well as other disorders (e.g., chronic fatigue syndrome, depression, ADHD)

(Carvalho et al., 2016; Hoenemeyer et al., 2018; Kaptchuk et al., 2010; Kleine-Borgmann et al., 2019; Sandler and Bodfish, 2007). In contrast to “classical” placebos, which are typically administered without patients’ knowledge, OLPs are given with full disclosure and consent, thereby avoiding ethical and legal concerns related to deception.

Numerous RCTs in migraine therapy report notable improvements in placebo arms (Speciali et al., 2010; Visser et al., 2004), highlighting the potential of placebo effects. Particularly striking are recently published studies on the intravenous administration of eptinezumab, a CGRP ligand antibody, for EM and CM (Ashina et al., 2020; Lipton et al., 2020). Both studies showed significant symptom reduction in the placebo groups. While most clinical trials have used deceptive placebos, Kam-Hansen et al. were the first to demonstrate positive effects of non-deceptive (i.e., open-label) placebo administration in the treatment of acute migraine attacks (Kam-Hansen et al., 2014). However, systematic studies on the effects of OLP in migraine—especially chronic migraine—are still lacking.

To make placebo responses clinically usable, it is necessary to predict an individual’s ability to develop placebo effects in a context-, organ system-, and disease-specific manner (Enck et al., 2013; Horing et al., 2014; Kaptchuk et al., 2008). Growing evidence suggests that genetic polymorphisms involved in dopaminergic and opioidergic signaling pathways may influence the magnitude of placebo responses (Hall et al., 2015, 2012). Furthermore, neuroimaging studies have shown structural and functional brain alterations in migraine patients (Bashir et al., 2013; Coppola et al., 2019), particularly in the descending pain modulatory system, which plays a key role in mediating placebo effects (Stein et al., 2012) and may therefore influence OLP effects in migraine patients (Liu et al., 2019, 2017). Identifying structural, functional, and cognitive preconditions—such as cognitive flexibility—is thus crucial to

pinpoint those patients who may particularly benefit from OLP interventions (Douw et al., 2016).

This randomized controlled trial aims to systematically investigate the effects of a 12-week open-label placebo treatment on the number of headache days (see definition of "headache day") in patients with EM and CM, in addition to treatment as usual (TAU). Secondary endpoints include the number of migraine days (see definition of "migraine day"), pain intensity, acute medication use, global impression of change, quality of life, tolerability, and treatment expectations and experiences. To systematically assess potential predictors of placebo responses in migraine patients, biological markers will also be measured in addition to psychometric parameters. These include cortisol and alpha-amylase awakening responses in saliva (as indicators of HPA axis and sympathetic nervous system activity) (Pruessner et al., 1997; Russell and Lightman, 2019; Stalder et al., 2016), the catechol-O-methyltransferase val158met polymorphism (Hall et al., 2012), as well as functional (resting-state fMRI) and structural (diffusion tensor imaging, DTI) brain connectivity.

2 Study Design and Objectives

2.1 Study Design

This is a multicenter, randomized, controlled clinical trial investigating the effects of open-label placebo (OLP) treatment in patients with episodic and chronic migraine, using a parallel-group design with two study arms: 1) OLP+TAU group, receiving a 12-week OLP intervention in addition to standard treatment (treatment as usual, TAU), and 2) TAU group, receiving standard treatment only, without any additional intervention.

Study initiation is planned for August 2020, with a target enrollment of 150 patients in total. The study is conducted at University Hospital Essen, Department of Neurology (main study center), and at the Migraine and Headache Clinic Königstein, Königstein im Taunus (secondary center). MRI data are collected exclusively at the main center in Essen. The trial was prospectively registered on October 9, 2020, in the German Clinical Trials Register (DRKS) under the registration number DRKS00021259.

2.2 Objectives

2.2.1 Primary Objective

The primary objective of this study is to evaluate the efficacy of an open-label placebo (OLP) treatment in patients with episodic migraine (EM) and chronic migraine (CM).

In accordance with the International Headache Society (IHS) guidelines for controlled trials of prophylactic treatment of chronic migraine in adults (Tassorelli et al., 2018), efficacy is assessed based on the number of headache days within a four-week period (see details below).

2.2.2 Secondary Objectives

The secondary objective is to investigate whether OLP treatment improves migraine-specific headaches, assessed by the number of migraine days in the past four weeks, as well as other patient-reported outcomes, including average pain intensity, quality of life, disability in daily life, global impression of change, tolerability, and the responder rate.

2.2.3 Exploratory Objectives

The exploratory objectives focus on identifying potential moderators and predictors of the open-label placebo (OLP) response. These include treatment expectation, pain-related fear, pain catastrophizing, personality traits, the cortisol and alpha-amylase awakening response in saliva, as well as the analysis of the catechol-O-methyltransferase (COMT) val158met polymorphism, and functional (resting-state fMRI) and structural (diffusion tensor imaging, DTI) brain connectivity.

Additionally, the study will assess subjective treatment expectations and experiences, indicators of psychological well-being (including anxiety, depression, somatization, and stress), and participants' willingness to initiate or continue OLP treatment after the study ends (within the TAU and OLP+TAU groups, respectively).

3 Study population

3.1 Inclusion Criteria

Inclusion criteria include an age of ≥ 18 years and a diagnosis of episodic or chronic migraine persisting for at least 12 months prior to screening—based on patient self-report and confirmed by a board-certified neurologist, according to the criteria of the International Classification of Headache Disorders (ICHD-III). Additionally, an average migraine frequency of ≥ 4 days per month over the three months prior to screening is required (see definition of “migraine day”). Participants must be capable of giving informed consent and have fluency in the German language. Following enrollment, a four-week baseline period is conducted to ensure medication stability prior to randomization.

3.2 Exclusion Criteria

Exclusion criteria include substance or alcohol abuse, a severe depressive episode, schizophrenia, suicidality, hypersensitivity or allergy to components of the placebo tablets, participation in another investigational drug trial within three months prior to enrollment, and the presence of acute or chronic pain syndromes other than migraine. Participants who voluntarily opt in to the optional MRI assessment must not suffer from claustrophobia and must not have implants or devices incompatible with MRI.

4 Study Intervention

4.1 Study Treatment and Arms

Eligibility screening takes place during the enrollment visit (Visit 0) and throughout the subsequent four-week baseline period. At Visit 0, all participants are asked to provide demographic data, current medications, a complete medical history, and information regarding alcohol and drug use, using a standardized in-house questionnaire.

At Visit 1, all participants watch an instructional video presenting general information about the placebo effect and findings from recent open-label placebo (OLP) studies (Kleine-Borgmann et al., 2019). Randomization into one of the two study arms then follows:

Intervention arm (OLP+TAU): Open-label placebo treatment in addition to stable standard therapy (treatment as usual, TAU)

Control arm (TAU): Continuation of stable standard therapy without any additional intervention.

All participants receive a standardized box (carton packaging), which is identical in both groups in terms of weight and sound when shaken.

OLP+TAU group: The box contains a labeled pill dispenser with 168 white placebo tablets (P tablets, 7 mm, Lichtenstein, Zentiva Pharma GmbH, Germany), an information leaflet stating that the tablets contain no active ingredient, and instructions to take one tablet twice daily for 12 weeks, in addition to their ongoing medication.

TAU group: The box contains no tablets but includes a letter informing the participant of their allocation to the control group, with no further measures required.

The white placebo tablets are composed of lactose monohydrate, cellulose, magnesium stearate (Ph. Eur.), and microcrystalline cellulose. Self-reported intake of placebo tablets is documented using a standardized headache diary.

4.2 Randomization

150 patients suffering from episodic and chronic migraine will be randomly allocated to one of two study arms according to an a priori randomization list generated by an independent member of the laboratory using R Studio (), RStudio Team (2020RStudio: Integrated Development for R, RStudio, Inc., Boston, MA; Version 1.2.5042). Patients will be asked to keep their treatment allocation confidential to ensure blinding of care providers, outcome assessors, and data analysts.

5 Endpoints and Assessments

5.1 Visit Schedule

Tab. 1. Visit Schedule

Difference to inclusion (in months)	**Visit 0 (Inclusion)** *0*	**Visit 1 (Baseline +R)[1]** *+1*	**Visit 2 (R+1m)** *+2m*	**Visit 3 (R+3m)** *+4m*	**Visit 4 (R+6m)** *+7m*	**Visit X (MRT)** *Unabh.*
Eligibility	X	X	-	-	-	-
Demography	X	-	-	-	-	-
**PRIMARY ENDPOINT**						
Headache days (4 weeks)	-	X	X	X	X	-
**SECONDARY ENDPOINTS**						
Migraine days (4 weeks)	-	X	X	X	X	-
Mean pain intensity (4 weeks)	-	X	X	X	X	-
Rescue medication days (4 weeks)	-	X	X	X	X	-
Global Impression of Change (PGIC)	-	-	X	X	X	-
Quality of life (SF-12)	-	X	X	X	X	-
Disability (PDI, HIT-6)	-	X	X	X	X	-
Side effects (GASE)	-	X	X	X	X	-
50%-Responder-rate	-	-	X	X	X	-
**EXPLORATIVE ENDPUNKTE**						
Functional brain connectivity (rsfMRI)	-	-	-	-	-	X[2]
Structural brain connectivity (DTI)	-	-	-	-	-	X[2]
Salivary awakening response (sAA)	X[3]	-	-	-	-	-
Genetics (COMT-polymorphism)	X	-	-	-	-	-
Treatment expectations (TEX-Q)	-	X	-	-	-	-
Treatment expectations and experiences (G-EEE)[4]	-	X	X	X	X	-

Difference to inclusion (in months)	**Visit 0 (Inclusion)** *0*	**Visit 1 (Baseline +R)[1]** *+1*	**Visit 2 (R+1m)** *+2m*	**Visit 3 (R+3m)** *+4m*	**Visit 4 (R+6m)** *+7m*	**Visit X (MRT)** *Unabh.*
Fear of pain (FPQ-III)	-	X	-	-	-	-
Pain catastrophizing (PCS)	-	X	-	-	-	-
Behavioral Approach System (BIS BAS)	-	X	-	-	-	-
Personality (BFI-10)	-	X	-	-	-	-
Somatization (SASS)	-	X	-	-	-	-
Depression, anxiety (STADI)	-	X	-	-	-	-
Stress (PSS)	-	X	-	-	-	-

R: Randomization, SF-12: Short Form Health Survey, PDI: Pain Disability Index, HIT-6: Headache Impact Test, PGIC: Patient Global Impression of Change, GASE: General Assessment of Side Effects, rsfMRT: Resting-State-Funktions-MRT, DTI: Diffusions-Tensor-Bildgebung, sAA: Salivary-Alpha-Amylase, COMT: Catechol-O-Methyltransferase, TEX-Q: Treatment Expectation Questionnaire, FPQ-III: Fear of Pain Questionnaire, PCS: Pain Catastrophizing Scale, BIS BAS: Behavioral Inhibition and Approach System, BFI-10: Short version of Big-Five-Inventory, SASS: Skala zur Erfassung somatischer Symptome, STADI: State-Trait-Angst-Depressions-Inventar, PSS: Perceived Stress Scale, OLP: Open-Label-Placebo, TAU: treatment as usual.

1 All assessments will be performed prior to randomization.
2 Facultative visit, only at primary study center.
3 Assessment prior to randomization.
4 In-house questionnaire
5 Post outcome assessments
6 After visit 4, if requested

5.2 Primary Endpoint

The primary endpoint is the change in the number of headache days from the four-week baseline period (Baseline) to Visit 3 (end of the 12-week treatment phase), as measured using a standardized headache diary (Tassorelli et al., 2018). Further details are illustrated in Figure 1. Data collection is conducted via online questionnaires (LimeSurvey, LimeSurvey GmbH, Hamburg) or on-site at the study center by a blinded study team member (e.g., for MRI or blood sampling).

5.2.1 Definitions

All definitions are based on the Guidelines for Controlled Trials of Preventive Treatment of Episodic Migraine in Adults (Tassorelli et al., 2018) and the International Classification of Headache Disorders, 3rd edition (ICHD-III) (Olesen, 2018).

5.2.1.1 Migraine Day

A migraine day is defined as a day with headache lasting at least 4 hours that fulfills criteria C and D of the ICHD-III for migraine without aura, or criteria B and C for migraine with aura, or the criteria for a probable migraine according to ICHD-III. Alternatively, a day is also counted as a migraine day if a headache is successfully treated with a triptan, ergotamine, or another migraine-specific acute medication. For the full ICHD-III criteria, see (Olesen, 2018).

5.2.1.2 Headache Day

A headache day is defined as a day with moderate to severe headache lasting at least 4 hours, or a day with a headache lasting at least 30 minutes that is successfully treated with an acute medication.

5.3 Secondary Endpoints

Secondary endpoints comprise

- Change in migraine days from Baseline to visit 3
- Change in mean pain intensity (numeric rating scale, 0 = no pain, 10 = worst pain)
- Change in rescue medication days in a 4-week-period (assessed by headache diary)
- Change in Pain Disability Index (PDI) (Tait et al., 1990) and Headache Impact Test (HIT-6) (Kosinski et al., 2003)
- Response proportions of Patient Global Impression of Change (PGIC) (Dodick et al., 2007)
- Change in quality of life based on Short Form Health Survey (SF-12) (Kosinski et al., 2003)
- Safety and tolerability of OLP assessed by General Assessment of Side Effects (GASE) (Rief et al., 2011)
- Responderrate, defined as proportion of patients with a ≥50 % reduction in headache days from Baseline to Visit 3.

5.4 Exploratory Endpoints

Exploratory endpoints comprise:

- Change in all primary and secondary endpoints from Baseline to Visit 2 (short-term effects) and from Baseline bis Visit 4 (long-term effects)
- Functional and structural brain connectivity (rsfMRT and DTI, optional Visit X)
- Cortisol- and sAA-awakening response between Visit 0 and Visit 1
- Blood sample at Visit 0 for PCR analysis of Catechol-O-Methyltransferase val158met-Polymorphisms

- Treatment expectation assessed by Treatment Expectation Questionnaire (TEX-Q) (Alberts et al., 2020)
- Fear of pain, assessed by Fear of Pain Questionnaire (FPQ-III) (Vambheim et al., 2017)
- Pain catastrophizing assessed by Pain Catastrophizing Scale (PCS) (Osman et al., 1997)
- Personality traits assessed by Behavioral Inhibition and Approach System (BIS/BAS) (Strobel et al., 2006) and Big Five Inventory (BFI-10) (Rammstedt and John, 2007)
- Anxiety and depression assessed by State-Trait-Angst-Depressions-Inventar (STADI) (Lothar Laux et al., 2013)
- Somatization assessed by Somatosensory Amplification Scale (SSAS) (Barsky et al., 1990)
- Sensitivity to stress assessed by Perceived Stress Scale (PSS) (Klein et al., 2016)

An in-house questionnaire (G-EEE: Generic Rating for Treatment Pre-experiences, Treatment Expectations, and Treatment Effects) is used to assess prior treatment experiences, expectations, and subjective evaluations of treatment effects over the course of the study. Visit 4 is designed to capture potential long-term effects of the OLP treatment on all primary and secondary endpoints in both the OLP+TAU and TAU groups. In addition, participants' motivations for study participation (assessed at Visit 0) and their assumptions about the mechanisms of action of OLP (assessed via an open-ended question at Visit 4) will be collected.

6 Statistical Considerations

6.1 Sample Size Calculation

We performed a sample size calculation using the statistical software G*Power (Faul et al., 2007). For our primary outcome, to reach a power of 0.9 with an alpha level of 0.05 and an effect size of f=0.2 (i.e., d=0.4), a total sample size of N=134 is needed. We decided for a sample size calculation based on our recent OLP trial investigating pain relief in chronic back pain with an effect size of d=0.44 (Kleine-Borgmann et al., 2019). However, other OLP trials report higher effect sizes (i.e., chronic low back pain: d=0.77 (Carvalho et al., 2016); irritable bowel syndrome: d=0.79 (Kaptchuk et al., 2010)). To account for a potential dropout rate of 10% we plan to enroll N=150 patients (N=75 patients per group).

6.2 Statistical Analysis Plan

The data analysis will be performed on basis of the general linear model considering repeated measures (mixed model). All main and interaction effects of the experimentally controlled factors *group* (between-group) and *time of measurement* (within-group) will be considered. The focus is on the interaction effect, which represents the differences in the change between both groups. This model will be equally applied to all outcome variables. Possible correlations between different outcome variables as well as possible predictive genetic factors and cortisol levels will be examined exploratively by extending the model by further linear cofactors. Imaging data will be analyzed performing correlation analyses between OLP effects and white matter (DTI) as well as potential changes in functional connectivity (rsfMRI), respectively.

Based on previous findings from patient samples suffering from chronic back pain (Carvalho et al., 2016; Kleine-Borgmann et al.,

2019), irritable bowel syndrome (Kaptchuk et al., 2010) and acute migraine attacks (Kam-Hansen et al., 2014) we expect a significant reduction of headache days in the OLP+TAU group compared to the TAU group over time (primary outcome). Also, we expect significant improvement in pain intensity, migraine days and parameters of psychological well-being (secondary and exploratory outcomes), and a reduced need for acute medication and side effects. We hypothesize that methionine/methionine homozygotes in the OLP+TAU group show stronger improvement in all outcomes compared to valine/valine homozygotes, and heterozygotes (Hall et al., 2015, 2012). Moreover, we hypothesize that higher awakening responses in salivary cortisol and sAA activity will be associated with weaker improvements in all outcomes and less susceptibility to OLP treatment. Furthermore, we will analyze functional and structural brain connectivity as potential predictors for exploratory purposes.

7 References

1. Alberts, J., Löwe, B., Glahn, M.A., Petrie, K., Laferton, J., Nestoriuc, Y., Shedden-Mora, M., 2020. Development of the generic, multidimensional Treatment Expectation Questionnaire (TEX-Q) through systematic literature review, expert surveys and qualitative interviews. BMJ Open 10, 36169. https://doi.org/10.1136/bmjopen-2019-036169

2. Ashina, M., Saper, J., Cady, R., Schaeffler, B.A., Biondi, D.M., Hirman, J., Pederson, S., Allan, B., Smith, J., 2020. Eptinezumab in episodic migraine: A randomized, double-blind, placebo-controlled study (PROMISE-1). Cephalalgia 40, 241–254. https://doi.org/10.1177/0333102420905132

3. Barsky, A.J., Wyshak, G., Klerman, G.L., 1990. The Somatosensory Amplification Scale and its relationship to hypochondriasis. J Psychiatr Res 24, 323–334. https://doi.org/10.1016/0022-3956(90)90004-A

4. Bashir, A., Lipton, R.B., Ashina, S., Ashina, M., 2013. Migraine and structural changes in the Brain: A systematic review and meta-analysis. Neurology. https://doi.org/10.1212/WNL.0b013e3182a6cb32

5. Carvalho, C., Caetano, J.M., Cunha, L., Rebouta, P., Kaptchuk, T.J., Kirsch, I., 2016. Open-label placebo treatment in chronic low back pain: A randomized controlled trial. Pain 157, 2766–2772. https://doi.org/10.1097/j.pain.0000000000000700

6. Coppola, G., Di Renzo, A., Petolicchio, B., Tinelli, E., Di Lorenzo, C., Parisi, V., Serrao, M., Calistri, V., Tardioli, S., Cartocci, G., et al., 2019. Aberrant interactions of cortical networks in chronic migraine: A resting-state fMRI study. Neurology 92, E2550–E2558. https://doi.org/10.1212/WNL.0000000000007577

7. Dodick, D.W., Silberstein, S., Saper, J., Freitag, F.G., Cady, R.K., Rapoport, A.M., Mathew, N.T., Hulihan, J., Crivera, C., Rupnow, M.F.T., et al., 2007. The impact of topiramate on health-related quality of life indicators in chronic migraine. Headache 47, 1398–1408. https://doi.org/10.1111/j.1526-4610.2007.00950.x

8. Douw, L., Wakeman, D.G., Tanaka, N., Liu, H., Stufflebeam, S.M., 2016. State-dependent variability of dynamic functional connectivity between frontoparietal and default networks relates to cognitive flexibility. Neuroscience 339, 12–21. https://doi.org/10.1016/j.neuroscience.2016.09.034

9. Enck, P., Bingel, U., Schedlowski, M., Rief, W., 2013. The placebo response in medicine: minimize, maximize or personalize? Nat Rev Drug Discov 12, 191–204. https://doi.org/10.1038/nrd3923

10. Faul, F., Erdfelder, E., Lang, A.G., Buchner, A., 2007. G*Power 3: A flexible statistical power analysis program for the social, behavioral, and biomedical sciences, in: Behavior Research Methods. Psychonomic Society Inc., pp. 175–191. https://doi.org/10.3758/BF03193146

11. Gallagher, R.M., Kunkel, R., 2003. Migraine medication attributes important for patient compliance: Concerns about side effects may delay treatment. Headache 43, 36–43. https://doi.org/10.1046/j.1526-4610.2003.03006.x

12. Hall, K.T., Lembo, A.J., Kirsch, I., Ziogas, D.C., Douaiher, J., Jensen, K.B., Conboy, L.A., Kelley, J.M., Kokkotou, E., Kaptchuk, T.J., 2012. Catechol-O-Methyltransferase val158met Polymorphism Predicts Placebo Effect in Irritable Bowel Syndrome. PLoS One 7, e48135. https://doi.org/10.1371/journal.pone.0048135

13. Hall, K.T., Loscalzo, J., Kaptchuk, T.J., 2015. Genetics and the placebo effect: The placebome. Trends Mol Med. https://doi.org/10.1016/j.molmed.2015.02.009

14. Hoenemeyer, T.W., Kaptchuk, T.J., Mehta, T.S., Fontaine, K.R., 2018. Open-Label Placebo Treatment for Cancer-Related Fatigue: A Randomized-Controlled Clinical Trial. Sci Rep 8, 2784. https://doi.org/10.1038/s41598-018-20993-y

15. Horing, B., Weimer, K., Muth, E.R., Enck, P., 2014. Prediction of placebo responses: A systematic review of the literature. Front Psychol 5, 1079. https://doi.org/10.3389/fpsyg.2014.01079

16. James, S.L., Abate, D., Abate, K.H., Abay, S.M., Abbafati, C., Abbasi, N., Abbastabar, H., Abd-Allah, F., Abdela, J., Abdelalim, A., et al., 2018. Global, regional, and national incidence, prevalence, and years lived with disability for 354 Diseases and Injuries for 195 countries and territories, 1990-2017: A systematic analysis for the Global Burden of Disease Study 2017, The Lancet. https://doi.org/10.1016/S0140-6736(18)32279-7

17. Kam-Hansen, S., Jakubowski, M., Kelley, J.M., Kirsch, I., Hoaglin, D.C., Kaptchuk, T.J., Burstein, R., 2014. Altered placebo and drug labeling changes the outcome of episodic migraine attacks. Sci Transl Med 6, 218ra5-218ra5. https://doi.org/10.1126/scitranslmed.3006175

18. Kaptchuk, T.J., Friedlander, E., Kelley, J.M., Sanchez, M.N., Kokkotou, E., Singer, J.P., Kowalczykowski, M., Miller, F.G., Kirsch, I., Lembo, A.J., 2010. Placebos without deception: a randomized controlled trial in irritable bowel syndrome. PLoS One 5, e15591. https://doi.org/10.1371/journal.pone.0015591

19. Kaptchuk, T.J., Kelley, J.M., Deykin, A., Wayne, P.M., Lasagna, L.C., Epstein, I.O., Kirsch, I., Wechsler, M.E., 2008. Do “placebo

responders” exist? Contemp Clin Trials 29, 587–595. https://doi.org/10.1016/j.cct.2008.02.002

20. Klein, E.M., Brähler, E., Dreier, M., Reinecke, L., Müller, K.W., Schmutzer, G., Wölfling, K., Beutel, M.E., 2016. The German version of the Perceived Stress Scale - psychometric characteristics in a representative German community sample. BMC Psychiatry 16, 159. https://doi.org/10.1186/s12888-016-0875-9

21. Kleine-Borgmann, J., Schmidt, K., Hellmann, A., Bingel, U., 2019. Effects of open-label placebo on pain, functional disability, and spine mobility in patients with chronic back pain: A randomized controlled trial. Pain 160, 2891–2897. https://doi.org/10.1097/j.pain.0000000000001683

22. Kosinski, M., Bayliss, M.S., Bjorner, J.B., Ware, J.E., Garber, W.H., Batenhorst, A., Cady, R., Dahlöf, C.G.H., Dowson, A., Tepper, S., 2003. A six-item short-form survey for measuring headache impact: The HIT-6™. Quality of Life Research 12, 963–974. https://doi.org/10.1023/A:1026119331193

23. Lipton, R.B., Bigal, M.E., Diamond, M., Freitag, F., Reed, M.L., Stewart, W.F., 2007. Migraine prevalence, disease burden, and the need for preventive therapy. Neurology 68, 343–349. https://doi.org/10.1212/01.wnl.0000252808.97649.21

24. Lipton, R.B., Goadsby, P.J., Smith, J., Schaeffler, B.A., Biondi, D.M., Hirman, J., Pederson, S., Allan, B., Cady, R., 2020. Efficacy and safety of eptinezumab in patients with chronic migraine: PROMISE-2. Neurology 94, e1365–e1377. https://doi.org/10.1212/WNL.0000000000009169

25. Liu, J., Ma, S., Mu, J., Chen, T., Xu, Q., Dun, W., Tian, J., Zhang, M., 2017. Integration of white matter network is associated with

interindividual differences in psychologically mediated placebo response in migraine patients. Hum Brain Mapp 38, 5250–5259. https://doi.org/10.1002/hbm.23729

26. Liu, J., Mu, J., Chen, T., Zhang, M., Tian, J., 2019. White matter tract microstructure of the mPFC-amygdala predicts interindividual differences in placebo response related to treatment in migraine patients. Hum Brain Mapp 40, 284–292. https://doi.org/10.1002/hbm.24372

27. Lothar Laux, Michael Hock, Ralf Bergner-Köther, Volker Hodapp, Karl-Heinz Renner, 2013. STADI - Das State-Trait-Angst-Depressions-Inventar.

28. Negro, A., Martelletti, P., 2019. Patient selection for migraine preventive treatment with anti-CGRP(r) monoclonal antibodies. Expert Rev Neurother. https://doi.org/10.1080/14737175.2019.1621749

29. Olesen, J., 2018. Headache Classification Committee of the International Headache Society (IHS) The International Classification of Headache Disorders, 3rd edition. Cephalalgia. https://doi.org/10.1177/0333102417738202

30. Osman, A., Barrios, F.X., Kopper, B.A., Hauptmann, W., Jones, J., O'Neill, E., 1997. Factor structure, reliability, and validity of the pain catastrophizing scale. J Behav Med 20, 589–605. https://doi.org/10.1023/A:1025570508954

31. Pruessner, J.C., Wolf, O.T., Hellhammer, D.H., Buske-Kirschbaum, A., Von Auer, K., Jobst, S., Kaspers, F., Kirschbaum, C., 1997. Free cortisol levels after awakening: A reliable biological marker for the assessment of adrenocortical activity. Life Sci 61, 2539–2549. https://doi.org/10.1016/S0024-3205(97)01008-4

32. Rammstedt, B., John, O.P., 2007. Measuring personality in one minute or less: A 10-item short version of the Big Five Inventory in English and German. J Res Pers 41, 203–212. https://doi.org/10.1016/j.jrp.2006.02.001

33. Rief, W., Barsky, A.J., Glombiewski, J.A., Nestoriuc, Y., Glaesmer, H., Braehler, E., 2011. Assessing general side effects in clinical trials: Reference data from the general population. Pharmacoepidemiol Drug Saf 20, 405–415. https://doi.org/10.1002/pds.2067

34. Russell, G., Lightman, S., 2019. The human stress response. Nat Rev Endocrinol. https://doi.org/10.1038/s41574-019-0228-0

35. Sandler, A.D., Bodfish, J.W., 2007. Open-label use of placebos in the treatment of ADHD: a pilot study. Child Care Health Dev 34, 104–110. https://doi.org/10.1111/j.1365-2214.2007.00797.x

36. Speciali, J.G., Peres, M., Bigal, M.E., 2010. Migraine treatment and placebo effect. Expert Rev Neurother. https://doi.org/10.1586/ern.10.8

37. Stalder, T., Kirschbaum, C., Kudielka, B.M., Adam, E.K., Pruessner, J.C., Wüst, S., Dockray, S., Smyth, N., Evans, P., Hellhammer, D.H., et al., 2016. Assessment of the cortisol awakening response: Expert consensus guidelines. Psychoneuroendocrinology. https://doi.org/10.1016/j.psyneuen.2015.10.010

38. Stein, N., Sprenger, C., Scholz, J., Wiech, K., Bingel, U., 2012. White matter integrity of the descending pain modulatory system is associated with interindividual differences in placebo analgesia. Pain 153, 2210–2217. https://doi.org/10.1016/j.pain.2012.07.010

39. Strobel, A., Beauducel, A., Debener, S., Brocke, B., 2006. Eine deutschsprachige Version des BIS/BAS-Fragebogens von Carver

und White. http://dx.doi.org/10.1024//0170-1789.22.3.216. https://doi.org/10.1024//0170-1789.22.3.216

40. Sussman, M., Benner, J., Neumann, P., Menzin, J., 2018. Cost-effectiveness analysis of erenumab for the preventive treatment of episodic and chronic migraine: Results from the US societal and payer perspectives. Cephalalgia 38, 1644–1657. https://doi.org/10.1177/0333102418796842

41. Tait, R.C., Chibnall, J.T., Krause, S., 1990. The Pain Disability Index: psychometric properties. Pain 40, 171–182. https://doi.org/10.1016/0304-3959(90)90068-O

42. Tassorelli, C., Diener, H.C., Dodick, D.W., Silberstein, S.D., Lipton, R.B., Ashina, M., Becker, W.J., Ferrari, M.D., Goadsby, P.J., Pozo-Rosich, P., et al., 2018. Guidelines of the International Headache Society for controlled trials of preventive treatment of chronic migraine in adults. Cephalalgia 38, 815–832. https://doi.org/10.1177/0333102418758283

43. Vambheim, S.M., Lyby, P.S., Aslaksen, P.M., Flaten, M.A., Åsli, O., Martinussen, L.M., 2017. The fear of pain questionnaire-III and the fear of pain questionnaire-short form: A confirmatory factor analysis. J Pain Res 10, 1871–1878. https://doi.org/10.2147/JPR.S133032

44. Visser, W.H., Winner, P., Strohmaier, K., Klipfel, M., Peng, Y., McCarroll, K., Cady, R., Lewis, D., Nett, R., Rizatriptan Protocol 059 and 061 Study Groups, 2004. Rizatriptan 5 mg for the acute treatment of migraine in adolescents: results from a double-blind, single-attack study and two open-label, multiple-attack studies. Headache 44, 891–9. https://doi.org/10.1111/j.1526-4610.2004.04171.x
