# Supplementary material for: Open-Label Placebos as Adjunct for the Preventive Treatment of Migraine: A Randomized Clinical Trial
Source: JAMA Netw Open. 2025 Oct 8;8(10):e2535739. doi: 10.1001/jamanetworkopen.2025.35739 (PMC12509028; doi:10.1001/jamanetworkopen.2025.35739)
Supplement: Supplement 3. — Data Sharing Statement [file jamanetwopen-e2535739-s003.pdf]

Data Sharing Statement

Kleine-Borgmann. Open-Label Placebos as Adjunct for the Preventive Treatment of Migraine. *JAMA Netw Open*. Published October 08, 2025. doi:10.1001/jamanetworkopen.2025.35739

Data
Additional Information: German Clinical Trial Register (DRKS), ID: DRKS00021259, URL: https://drks.de/search/en/trial/DRKS00021259
Data available: Yes
Data types: Deidentified participant data, Data dictionary
How to access data: Due to sensitivity, the data supporting this study's findings are not openly available but are available from the corresponding author upon reasonable request (julian.kleine-borgmann@uk-essen.de). The data are located in controlled access data storage at University Medicine Essen, Essen, Germany.
When available: With publication

Supporting Documents
Document types: None

Additional Information
Who can access the data: Due to sensitivity, the data supporting this study's findings are not openly available but are available from the corresponding author upon reasonable request (julian.kleine-borgmann@uk-essen.de). The data are located in controlled access data storage at University Medicine Essen, Essen, Germany.
Types of analyses: Specified purposes, e.g., secondary analyses/meta-analyses.
Mechanisms of data availability: Data are available from the corresponding author upon reasonable request (julian.kleine-borgmann@uk-essen.de)
